# Effects of Melatonin Dose Regimens on Follicular Development and Oocyte Developmental Competence in FSH-Superovulated Ewes: Redox and Molecular Responses

**DOI:** 10.3390/antiox15101346

**Published:** 2026-10-18

**Authors:** Aikebaier Aihemaiti, Alimire Niyazi, Kuanfeng Zhu, Weijian Li, Depeng Yin, Bingyuan Wang, Xuguang Wang, Lu Zhang, Guoshi Liu

**Affiliations:** 1 College of Animal Science, Xinjiang Agricultural University, Urumqi 830091, China; 2 National Engineering Laboratory for Animal Breeding, Key Laboratory of Animal Genetics and Breeding of the Ministry of Agriculture, Beijing Key Laboratory for Animal Genetic Improvement, College of Animal Science and Technology, China Agricultural University, Beijing 100193, China

**Keywords:** melatonin, oxidative stress, mitochondria, follicular development, oocyte

## Abstract

Oxidative stress impairs gamete development and embryonic competence in female mammals. We compared four melatonin (MT) dose regimens (vehicle control and 20, 40, and 80 mg per injection) in FSH-superovulated ewes during in vivo ovum pick-up. The 40 mg regimen significantly reduced follicular atresia and increased high-quality oocytes and transferable blastocysts. Pregnancy and lambing rates after in vivo embryo transfer were numerically higher in the 40 mg group but did not reach statistical significance. The favorable responses observed with the 40 mg regimen were not maintained at the 80 mg dose. Oocyte transcriptomics and follicular fluid metabolomics revealed multilevel molecular responses associated with MT treatment. Systemically, 40 mg MT was associated with changes in serum reproductive hormones, including significant increases in MT, E2, and IGF-1. At the cellular and molecular levels, MT treatment was associated with changes in follicular development and oocyte quality, together with altered redox status and candidate changes in energy metabolism; MT1-related signaling was identified as a candidate pathway. MT treatment was associated with increased MT1 and Ki-67 expression in granulosa cells, an increased Bcl-2/Bax ratio, and reduced follicular fluid ROS and MDA levels, indicating improved redox homeostasis and granulosa cell proliferation. MT treatment was associated with altered redox status, including significantly increased GSH and reduced intracellular ROS, while ATP and mitochondrial superoxide showed non-significant changes. Hypoxia-inducible factor 1 (HIF-1) signaling and the tricarboxylic acid (TCA) cycle were identified as candidate pathways potentially associated with follicular energy–redox remodeling. These observations provide a basis for further investigation of MT dose regimens to mitigate disadvantages associated with FSH superovulation in ewes and other species.

## 1. Introduction

Mammalian follicular reserve and oocyte developmental competence are core determinants of female reproductive function. Disruption of redox homeostasis triggers a persistent cascade of damage [1]: excessive reactive oxygen species (ROS) induces follicular atresia and granulosa cell apoptosis, disrupts mitochondria, impairs the tricarboxylic acid (TCA) cycle and adenosine triphosphate (ATP) synthesis, and blocks oocyte cytoplasmic maturation [2,3,4]. In large-scale livestock production employing follicle-stimulating hormone (FSH) superovulation and in vitro embryo production, exogenous gonadotropins readily disturb ovarian redox balance, causing extensive follicular atresia [5,6] and reduced oocyte yield [7], which severely constrains efficient sheep breeding. Accordingly, elucidating endogenous antioxidant pathways to stabilize redox homeostasis in the female reproductive tract has emerged as a key research priority at the intersection of animal reproduction and antioxidant biology. Melatonin (MT), synthesized by the pineal gland and locally produced in ovarian tissue via autocrine secretion, regulates hypothalamic–pituitary–ovarian axis function through endocrine pathways [8]. High levels of MT have been detected in follicular fluid, and its receptors MT1 and MT2 are expressed in granulosa cells and oocytes, enabling MT to directly target the ovary and coordinate oxidative stress and apoptosis [9,10]. In vitro co-culture studies have confirmed that exogenous MT in maturation medium reduces ROS accumulation in cumulus–oocyte complexes (COCs), elevates glutathione (GSH) reserves, and alleviates oxidative stress-mediated gamete damage [11]. Moreover, in vivo studies in mice [12] and cattle [13,14] have shown that MT reduces atretic follicles, promotes follicle recruitment, and improves blastocyst development.

Short-term in vitro MT treatment cannot replicate the complete endogenous endocrine network of live animals or the long-term physiological shifts induced by FSH superovulation; consequently, findings from in vitro studies cannot be directly extrapolated to in vivo sheep breeding [15,16,17]. Furthermore, most published in vivo studies have used rodent models, which have short estrous cycles and hormonal pathways distinct from those of Hu sheep ewes, and most have used only a single dose without graded comparisons, making it difficult to systematically characterize the regulatory features of different MT doses on follicular development. In addition, most studies have focused on a single oxidative stress parameter or in vitro embryo phenotype, with very few integrating ovarian histopathology, granulosa cell proliferation/apoptosis, temporal changes in endogenous hormones, follicular fluid microenvironment, or oocyte organelle function for multi-dimensional validation. Similarly, few have combined oocyte transcriptomics with follicular fluid metabolomics for layered mechanistic dissection, leaving the molecular pathways governing endogenous antioxidant regulation in ruminant ovaries largely undefined. More importantly, existing studies have mostly relied on in vitro embryonic parameters and lack in vivo reproductive outcome data, such as pregnancy and lambing rates after embryo transfer; whether short-term follicular-phase MT intervention can genuinely improve livestock reproductive efficiency thus remains unverified [18]. Importantly, to our knowledge, such a comprehensive in vivo multi-gradient MT treatment system combined with multi-phenotypic detection, dual-omics profiling, and embryo-transfer-based reproductive outcome assessment has not been previously reported in adult ruminants.

Our group previously reported that subcutaneous MT slow-release implants applied to both donors and recipients in Small-Tailed Han sheep significantly increased corpus luteum counts and recovered embryo yields, and further improved post-transfer pregnancy and lambing rates [19]. In sika deer, our team also found MT implant treatment elevated serum gonadotropin levels and corpus luteum number, though embryo number only showed a non-significant upward trend and delayed embryonic development was observed [20]. However, those prior studies adopted sustained-release implants with MT exposure for both donors and recipients. Whether comparable reproductive benefits could be achieved by intermittent subcutaneous MT injection restricted only to donor ewes during the follicular phase remained unclear, which prompted the current study.

Given these gaps, several key questions remain unanswered in adult Hu sheep ewes: whether different MT dose regimens exert distinct effects on follicular development and which tested regimen is most favorable; whether the mitochondrial protective and antioxidant effects observed in vitro can be stably reproduced in the intact in vivo endocrine environment; and whether improvements in oocyte quality associated with MT treatment translate into tangible benefits in pregnancy and lambing after embryo transfer.

It is well established that oocytes can synthesize MT, with mitochondria as the primary synthesis site, and that locally produced MT acts through both receptor-dependent and receptor-independent pathways to reduce ROS, enhance mitochondrial electron transport chain function, augment oxidative phosphorylation, and increase ATP production [21,22]. However, controversial findings have been reported. For example, in bovine oocytes, MT combined with carnosine during in vitro maturation (IVM) synergistically improves blastocyst yield and total cell number per embryo, with concomitant reduction in ROS [23], whereas MT supplementation during the pre-maturation phase paradoxically elevates intracellular ROS and downregulates PLAC8, a key embryonic development gene [24]. These discrepancies indicate that MT’s biological effects are highly dependent on species background, administration window, and treatment duration, and that in vitro models alone cannot recapitulate true in vivo regulatory patterns.

Based on these considerations, we proposed two primary hypotheses: that different MT dose regimens would differ in their effects on follicular development and oocyte developmental competence in ewes, and that MT treatment would be accompanied by changes in redox homeostasis and mitochondrial function in oocytes. This study was not designed to establish a causal mechanism or to define an optimal dose. Instead, it was designed to compare the tested MT regimens across multiple levels, including ovarian, cellular, endocrine, and molecular responses.

To address these gaps and controversies, this study used FSH-synchronized superovulated adult Hu sheep ewes as an in vivo model and established a multi-dose subcutaneous MT administration system to compare the tested MT regimens for ovarian development and gamete maturation. Moving beyond single-parameter assays and simplified in vitro models, we integrated a multi-dimensional system encompassing tissue morphology, cell proliferation/apoptosis, endocrine microenvironment, organelle function, and reproductive phenotypes, combined with oocyte transcriptomics and follicular metabolomics to identify core regulatory molecules. Our objective was to compare the tested MT regimens and to identify candidate molecular and redox pathways associated with their effects on follicular development and oocyte quality.

This study is anchored in a framework that cross-validates multi-dimensional physiological phenotypes with dual-omics molecular data, focusing on the potential associations of redox homeostasis with MT’s effects on follicular atresia, follicular reserve, and oocyte developmental competence, thereby contributing to the understanding of endogenous antioxidant regulation in ruminant ovarian gamete development. The multi-phenotype, dual-omics in vivo data generated here provide a theoretical basis for further investigation of ovarian oxidative damage and oocyte mitochondrial metabolism in mammals, as well as for identifying candidate regulatory targets in gamete development.

## 2. Materials and Methods

### 2.1. Animals

Healthy adult Hu sheep ewes aged 1–2 years were selected. The majority had no history of previous lambing. All ewes were examined before inclusion and confirmed to be healthy, with normal reproductive history, regular estrous cycles and no signs of reproductive diseases. The study was conducted between March 2025 and May 2026. All ewes were from the same flock with relatively uniform body weight. Body weight was recorded for 146 ewes (mean ± SD: 42.4 ± 3.6 kg; range: 37.0–51.5 kg). All animals were housed under standardized feeding conditions with identical diets and ad libitum access to water, under natural light/dark conditions. Controlled internal drug release (CIDR) progesterone vaginal inserts were used for estrus synchronization, and the day of CIDR insertion was defined as Day 0. From Day 9 to Day 11, follicle-stimulating hormone (FSH) was administered intramuscularly twice daily (08:00 and 20:00) in descending doses: Day 9, 90 IU × 2; Day 10, 65 IU × 2; Day 11, 45 IU × 2; for a total dose of 400 IU, following a standardized superovulation protocol previously described by our group [25].

Melatonin (MT) stock solution was prepared with anhydrous ethanol:sterile normal saline = 7:3 (*v*/*v*). Briefly, MT powder was fully dissolved in anhydrous ethanol before constant volume adjustment to 100 mL to prepare reserve solutions at concentrations of 10, 20 and 40 mg/mL. Each ewe received a fixed subcutaneous injection of 2 mL of the corresponding working solution to establish three MT dosage groups: 20, 40, and 80 mg per injection, corresponding to cumulative doses of 120, 240, and 480 mg per ewe over the six-injection regimen. The control group was injected with 2 mL blank solvent of identical ethanol-saline ratio without MT. Subcutaneous MT injections were administered in parallel with each FSH administration (six injections). A fixed-dose regimen was adopted, consistent with previous studies of melatonin superovulation in sheep and other ruminants [19,20,25,26,27]. CIDR inserts were removed simultaneously with the final FSH injection. Laparotomy was conducted 12 h after CIDR withdrawal to collect ovarian tissues, follicles, oocytes and follicular fluid samples.

This experiment contained two independent modules: dosage screening and multi-dimensional response validation, utilizing completely separate populations of adult Hu sheep ewes to avoid sample cross-contamination. A total of 231 Hu sheep ewes were used in the whole trial. For the gradient dosage test, 96 ewes were completely randomly divided into four groups (0, 20, 40, and 80 mg MT) only for statistical analysis of follicle, oocyte and early embryonic indices. Independent animal cohorts were arranged for response detection. Ovarian histology and serum hormone detection were performed using 26 ewes with 13 individuals per group, and 18 ewes (9 per group) were used for oocyte mitochondrial fluorescence measurement. These H&E, immunohistochemistry, and serum hormone analyses were performed on subsets of the 26 ewes described above. For ovarian histology, six ewes per group were selected. For H&E staining and follicle counting, one ewe in the 40 mg MT group was excluded due to poor section quality, leaving five ewes in this group. For immunohistochemistry, all six ewes per group were included. For serum hormone analysis, seven ewes per group were included. A total of 16 donor ewes (8 per group) and 53 recipient ewes (26 in the control group, 27 in the 40 mg MT group) were prepared for embryo transfer experiments. Eight ewes (4 per group) were applied for oocyte transcriptome sequencing, and 14 ewes (six in the control group and eight in the 40 mg MT group) were subjected to follicular fluid metabolomics analysis, with six valid samples retained for each group after quality control. All follicular fluid samples were aliquoted into two portions for separate determination of hormonal and oxidative stress biomarkers. A detailed flow diagram of animal allocation, analysis, and exclusion is provided in Figure S1 (Supplementary File S1).

### 2.2. Reagents

Complete culture media for IVM, IVF, and embryo culture were purchased from IVF Bioscience (Falmouth, UK). Fluorescent probes for ROS detection and mitochondrial staining were obtained from Invitrogen (Carlsbad, CA, USA). Melatonin (M5250) was purchased from Sigma-Aldrich (St. Louis, MO, USA). CIDR devices were obtained from Zoetis (New York, NY, USA). ELISA kits for hormone and oxidative stress detection were purchased from Nanjing Jiancheng Bioengineering Institute (Nanjing, China); RNA extraction and qPCR kits were obtained from Tiangen Biochemical Technology Co., Ltd. (Beijing, China). Primary antibodies against Ki-67 (GB111499), Bax (GB114122), Bcl-2 (GB113375), FSH-R (GB11275-1), and MT1 (GB113284), as well as the HRP-conjugated secondary antibody (GB23303) and DAB chromogenic kit (G1212-200T), were purchased from Servicebio (Wuhan, China).

### 2.3. Oocyte In Vitro Maturation (IVM), In Vitro Fertilization (IVF), and Embryo In Vitro Culture (IVC)

At 12 h after CIDR removal, cumulus–oocyte complexes (COCs) were collected via laparotomy. The number of punctured follicles, recovered oocytes and usable oocytes were recorded, and two calculation indices were defined as follows: oocyte recovery rate = Number of recovered oocytes/Number of punctured follicles × 100%; usable oocyte rate = Number of usable oocytes/Number of recovered oocytes × 100%.

Oocytes were classified following the grading standard reported by Alvarez et al. [21]: Grade A, oocytes surrounded by ≥3 compact cumulus layers with homogeneous cytoplasm; Grade B, oocytes covered by ≥1 compact cumulus layer with relatively uniform cytoplasm; Grade C, oocytes with sparse cumulus cells or denuded cytoplasm of uniform morphology; Grade D, degenerated oocytes with irregular cytoplasmic shape. Only Grade A and Grade B COCs were subjected to subsequent experiments. All oocyte classification work was completed by the same researcher to reduce human error.

Qualified COCs were rinsed with BO-HEPES solution and cultured in BO-IVM medium at 38.5 °C under a humidified atmosphere with 5% CO_2_ for 24 h. After in vitro maturation, cumulus cells were stripped using 0.2% hyaluronidase, and oocytes were transferred into BO-IVF fertilization medium. Fresh ram semen was purified via the swim-up method, and the final sperm concentration was adjusted to 1 × 10^5^/mL, followed by 24 h co-incubation to complete fertilization. Fertilized oocytes were washed and transferred into BO-IVC one-step medium for continuous embryo culture.

Cleavage rate was counted at 48 h post-culture, and total blastocyst numbers were recorded on Days 6, 7 and 8. To guarantee consistent embryonic developmental stages, only morphologically normal Day 7 blastocysts were subjected to Hoechst 33,342 staining for total cell counting. Blastocysts were selected for staining at the group level: from each of the first four experimental batches, five blastocysts per treatment group were randomly selected, yielding a total of 20 blastocysts per group. Oocytes were pooled within each treatment group for IVM, IVF, and IVC, and individual donor origin was not recorded.

All blastocysts collected on Day 6, regardless of expansion status, were used for embryo transfer to recipient ewes. Within each treatment group, oocytes were pooled for IVM, IVF, and IVC, and blastocysts were collected at the group level rather than by individual donor ewe. Consequently, individual donor-to-embryo-to-recipient mapping was not recorded. Each synchronized recipient ewe received a single Day-6 blastocyst via surgical embryo transfer (single-embryo transfer).

### 2.4. Ovarian Tissue Hematoxylin and Eosin (H&E) Staining

Ovarian tissues were fixed in 4% paraformaldehyde, dehydrated through graded ethanol, cleared in xylene, and embedded in paraffin. Sections were cut at 5 μm thickness and stained with H&E. The numbers and proportions of primordial, growing, and atretic follicles in each section were counted using Saiviewer 2.2.4 software, with at least three sections per group used for quantitative analysis. Six ewes per group were used for H&E staining; one ewe in the 40 mg MT group was excluded due to poor section quality, leaving five ewes in this group for follicle counting.

### 2.5. Ovarian Tissue Immunohistochemical Staining

Paraffin sections were deparaffinized, rehydrated, and subjected to antigen retrieval by heating in citrate buffer. Endogenous peroxidase activity was blocked by incubation in 3% H_2_O_2_ at room temperature for 10 min, followed by blocking with 1% BSA at room temperature for 1 h. Sections were then incubated with primary antibodies against Ki-67 (1:500), Bax (1:400), Bcl-2 (1:500), FSH-R (1:200), and MT1 (1:1000) at 4 °C overnight, followed by HRP-conjugated goat anti-rabbit IgG (H + L) secondary antibody (1:200) incubation at room temperature, DAB chromogenic development, and counterstaining with hematoxylin. All staining procedures were performed under identical conditions.

Morphologically intact growing follicles were photographed under 200× magnification and subjected to semi-quantitative analysis using Saiviewer imaging software. The follicle interior was manually delineated as the region of interest (ROI) to exclude stromal interference. MT1, FSH-R, Bcl-2, and Bax were quantified as average optical density (AOD), while Ki-67 was expressed as the percentage of positive granulosa cell nuclei to reflect proliferative activity. For each Hu sheep ewe, at least five follicles per ovary were randomly selected. For each marker, the measured values (AOD for MT1, FSH-R, Bcl-2, and Bax; percentage of positive granulosa cells for Ki-67) were first averaged within each ovary, and the left and right ovarian means were then averaged to obtain a single value per ewe. The ewe was used as the experimental unit for statistical comparisons. Six ewes per group were used for immunohistochemical staining.

### 2.6. Fluorescence Detection of Oocyte Redox Status and Mitochondrial Function

Denuded oocytes from the control and 40 mg MT groups were collected. Four fluorescent probes were used to detect intracellular ROS, GSH, mitochondrial superoxide (mtO_2_^−^), and relative ATP levels. All fluorescence images were analyzed using ImageJ 1.54 software, with background subtraction and quantification of mean fluorescence intensity.

ROS and GSH were labeled with H_2_DCFDA (Invitrogen, #D399) and CellTracker Blue (Invitrogen, #C12881), respectively. Oocytes were incubated with 20 μmol/L fluorescent probe at 38.5 °C, 5% CO_2_ humid dark environment for 30 min. After PBS washing, sequential imaging was performed with FITC and DAPI channels. Mitochondrial superoxide was detected using 5 μmol/L MitoSOX Red (Invitrogen, #M36008) under the same culture conditions in the dark for 20 min, followed by washing and imaging. Relative ATP content was measured using the BODIPY FL ATP probe (Invitrogen, #A12410) according to a previously established method [28]: oocytes were fixed in 4% paraformaldehyde at room temperature for 1 h, incubated with 0.5 μmol/L probe in the dark for 1 h, washed, mounted, and imaged under a Nikon TE2000-S (Tokyo, Japan) upright fluorescence microscope.

Oocytes were obtained from 18 ewes (9 per group). Microscope exposure parameters were kept consistent across all samples during imaging.

### 2.7. Determination of Endocrine and Oxidative Biomarkers in Serum and Follicular Fluid

Blood samples were collected from the jugular vein before CIDR insertion (Day 0) and each morning before FSH injection (07:30–08:00) during superovulation treatment (Days 9–12). After standing at room temperature for stratification, serum was separated by centrifugation, aliquoted, and stored at −80 °C for subsequent analysis. ELISA kits were used to dynamically quantify serum concentrations of MT, estradiol (E_2_), luteinizing hormone (LH), FSH, progesterone (P_4_), insulin-like growth factor 1 (IGF-1), growth hormone (GH), prolactin (PRL), and anti-Müllerian hormone (AMH).

Follicular fluid was collected during laparotomy on D12, centrifuged to remove cellular debris, and the supernatant was aliquoted and stored at −80 °C. ELISA and fluorometric assay kits were used to measure follicular MT, E_2_, P_4_ and IGF-1 levels, together with total antioxidant capacity (T-AOC), GSH, ROS and malondialdehyde (MDA).

### 2.8. Transcriptome Sequencing of Oocytes

Four biological replicates were collected from both the control and 40 mg MT groups, with 10 mature MII-stage oocytes per replicate. Immediately after collection, oocytes were lysed thoroughly in lysis buffer containing RNase inhibitors and stored at −80 °C before transportation on dry ice. Smart-seq technology was applied for library construction and high-throughput sequencing.

Raw sequencing data underwent quality control to obtain clean reads, which were aligned to the sheep reference genome (Oar_v4.0) using HISAT2 (v2.1.0). Gene expression levels were counted via htseq-count (v0.11.2). Differentially expressed genes (DEGs) were screened with DESeq2 software (v1.22.2)after normalization based on BaseMean values; the negative binomial distribution test combined with the Benjamini–Hochberg correction was adopted to calculate adjusted *p*-values (padj). The screening thresholds were set as |log_2_FC| ≥ 1 and padj < 0.05, with a fold change ≥ 1.5 as an auxiliary criterion.

All bioinformatic analyses and visualization plotting were completed on the OE Biotech Cloud Platform (https://cloud.oebiotech.com/task/, accessed on 15 July 2026), which performs statistical analysis and graph drawing based on the R programming language (https://www.r-project.org/, accessed on 10 August 2026). Gene Ontology (GO) annotation and Kyoto Encyclopedia of Genes and Genomes (KEGG) pathway enrichment analyses were performed on the screened DEGs, with the hypergeometric distribution algorithm coupled with Fisher’s exact test to assess enrichment significance. A protein–protein interaction (PPI) network was constructed using the STRING database (v12.5; https://cn.string-db.org, accessed on 15 August 2026) with a minimum interaction score of 0.400 (medium confidence) based on the screened DEGs. Hub genes were defined as the top-ranked nodes by degree using the network analysis function in Cytoscape (v3.9.1).

### 2.9. Quantitative Real-Time PCR (qRT-PCR) Technical Confirmation

cDNA from the same oocyte samples used for transcriptome library construction was used for qRT-PCR technical confirmation, not independent biological validation. Eleven of the 12 candidate genes were selected from the hub genes of the PPI network, and MTNR1A was selected because it was a DEG encoding the melatonin receptor, although it was not connected in the STRING network. Amplification reactions were carried out in a total volume of 20 μL using the SuperReal PreMix Plus kit (TIANGEN, Beijing, China, #FP205). Each reaction mixture contained 2 μL cDNA template, 10 μL 2× SuperReal PreMix Plus, 0.6 μL forward primer (10 μmol/L), 0.6 μL reverse primer (10 μmol/L), and nuclease-free water to reach the final volume.

Amplification was performed on a ViiA 7 Real-Time PCR System (Applied Biosystems, Foster city, CA, USA) following the thermal profile: an initial pre-denaturation step at 95 °C for 15 min, followed by 40 cycles of denaturation at 95 °C for 10 s and combined annealing-extension at 60 °C for 32 s. Melting curve analysis was conducted after amplification to confirm the specificity of PCR products. The relative gene expression levels were calculated using the 2^−^ΔΔCt method, with β-actin selected as the internal reference gene. Detailed primer sequences and corresponding annealing temperatures are summarized in Table 1.

### 2.10. Untargeted LC-MS/MS Metabolomic Analysis of Follicular Fluid

Six follicular fluid samples per group (control vs. 40 mg MT) were retained after QC screening. Briefly, 50 μL follicular fluid was mixed with 150 μL pre-cooled methanol–acetonitrile (2:1, *v*/*v*) spiked with internal standards, followed by thorough vortexing, 10 min ice-bath sonication, and overnight incubation at −40 °C. The mixture was centrifuged at 12,000 rpm, 4 °C for 20 min, and supernatants were collected for injection. Pooled QC samples were prepared by mixing equal volumes of all sample extracts.

Metabolite profiling was carried out on a Waters ACQUITY UPLC I-Class Plus coupled to a Thermo QE HF HR-MS. Separation used an ACQUITY UPLC HSS T3 column (100 mm × 2.1 mm, 1.8 μm) held at 45 °C, with a flow rate of 0.35 mL/min and 5 μL injection volume. Mobile phases were 0.1% aqueous formic acid (A) and pure acetonitrile (B). A HESI ion source was used; Full MS/dd-MS^2^ (TOP 10) spectra were acquired in both positive and negative modes to record primary and secondary fragments.

Raw MS data underwent baseline filtering, peak detection, retention time alignment, matching and normalization to yield a standardized quantification matrix. Metabolites were annotated and quantified against public databases. Differential metabolites were defined by VIP ≥ 1, |log_2_FC| ≥ log_2_(1.2), and *p* < 0.05, with KEGG enrichment conducted at *p* ≤ 0.05. Transcriptomic and metabolomic samples originated from separate ewe cohorts; integrated multi-omics correlation analysis was not performed.

### 2.11. Statistical Analysis

All experiments included ≥3 independent biological replicates; data were expressed as mean ± standard error of the mean (SEM). The experimental unit was the individual ewe for all endpoints, except for blastocyst total cell number, which was analyzed using a linear mixed model (LMM) with treatment as a fixed effect and experimental batch as a random effect to account for the nested structure of blastocysts within batches. GraphPad Prism 11.0.0 was used for plotting.

For ovarian morphological parameters (length, width, thickness, weight, and volume), measurements from the left and right ovaries were averaged for each ewe to obtain a single bilateral mean per animal. These bilateral means were compared using independent-samples t-tests and are presented as mean ± SEM.

Multi-group comparisons across four MT doses adopted a randomized block design with GLM (treatment as fixed effect, batch as block effect), followed by Bonferroni post hoc tests. For oocyte fluorescence intensity data (ATP, GSH, MitoSOX, and ROS), a linear mixed model (LMM) with treatment as a fixed effect and ewe as a random effect was applied to all individual oocyte measurements to account for the nested structure of multiple oocytes within each ewe. All other two-group datasets (ovarian histology, granulosa cell protein expression, follicular fluid biochemical indices, and qRT-PCR) were analyzed by two-tailed Welch’s *t*-tests or Mann–Whitney U tests based on normality. Time-series serum hormones were evaluated via two-way repeated-measures ANOVA with treatment as a between-subject factor and time as a within-subject factor, including the treatment × time interaction. Sphericity was assessed using Mauchly’s test, and the Greenhouse-Geisser correction was applied when sphericity was violated, followed by Šidák’s multiple comparison test.

Embryo transfer pregnancy and lambing rates were categorical data with small sample sizes, analyzed using two-sided Fisher’s exact test, with relative risk (RR) and 95% confidence intervals (CIs) calculated. For embryo transfer outcomes, the recipient ewe was the experimental unit. Because individual donor-to-recipient mapping was not retained, these analyses cannot account for potential clustering by donor ewe. For transcriptomic and metabolomic differential omics data, the Benjamini–Hochberg method corrected multiple testing, with adjusted *p*-values (padj/FDR) for significance judgment. Significance markers: * *p* < 0.05, ** *p* < 0.01, *** *p* < 0.001; *p* > 0.05 = non-significant (ns).

## 3. Results

### 3.1. Effects of MT Treatment on Follicle Number, Oocyte Recovery, and Embryo Development in Sheep

To determine the impacts of varying MT doses on ovarian follicle abundance and oocyte retrieval efficiency, bilateral ovarian follicle counts and oocyte-related indices were compared across the four experimental groups.

**Figure 1:**
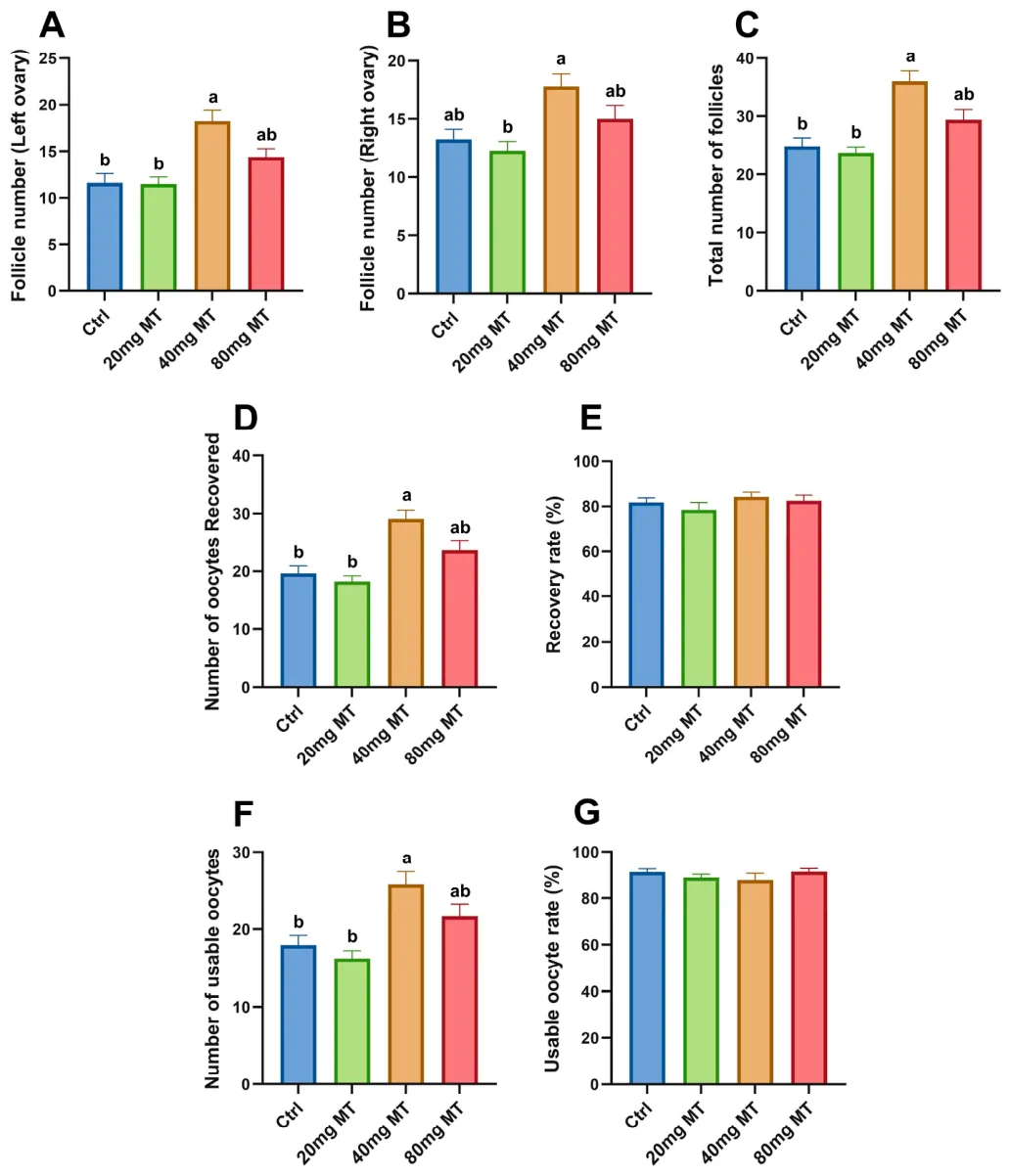
Effects of subcutaneous injection with different MT doses on ovarian follicle counts and oocyte retrieval indices in ewes (n = 24 per group). (**A**) Follicle number of the left ovary. (**B**) Follicle number of the right ovary. (**C**) Total follicle number of bilateral ovaries. (**D**) Number of recovered oocytes. (**E**) Oocyte recovery rate. (**F**) Number of usable oocytes. (**G**) Usable oocyte rate. Data are presented as mean ± SEM. Columns with different lowercase letters (a, b) indicate significant differences (*p* < 0.05) by Bonferroni post hoc test. Columns with the same letter indicate no significant difference (*p* > 0.05).

The results showed that the left, right, and total ovarian follicle counts in the 40 mg MT group were significantly higher than those in the control and 20 mg MT groups (*p* < 0.001; Figure 1A–C).

For oocyte retrieval indices, the numbers of recovered oocytes and usable oocytes were significantly higher in the 40 mg MT group than in the control and 20 mg MT groups (*p* < 0.001; Figure 1D,F). No statistically significant differences were observed for oocyte recovery rate or usable oocyte rate among the four groups (*p* > 0.05; Figure 1E,G).

### 3.2. Effects of MT on In Vitro Oocyte Developmental Competence

**Figure 2:**
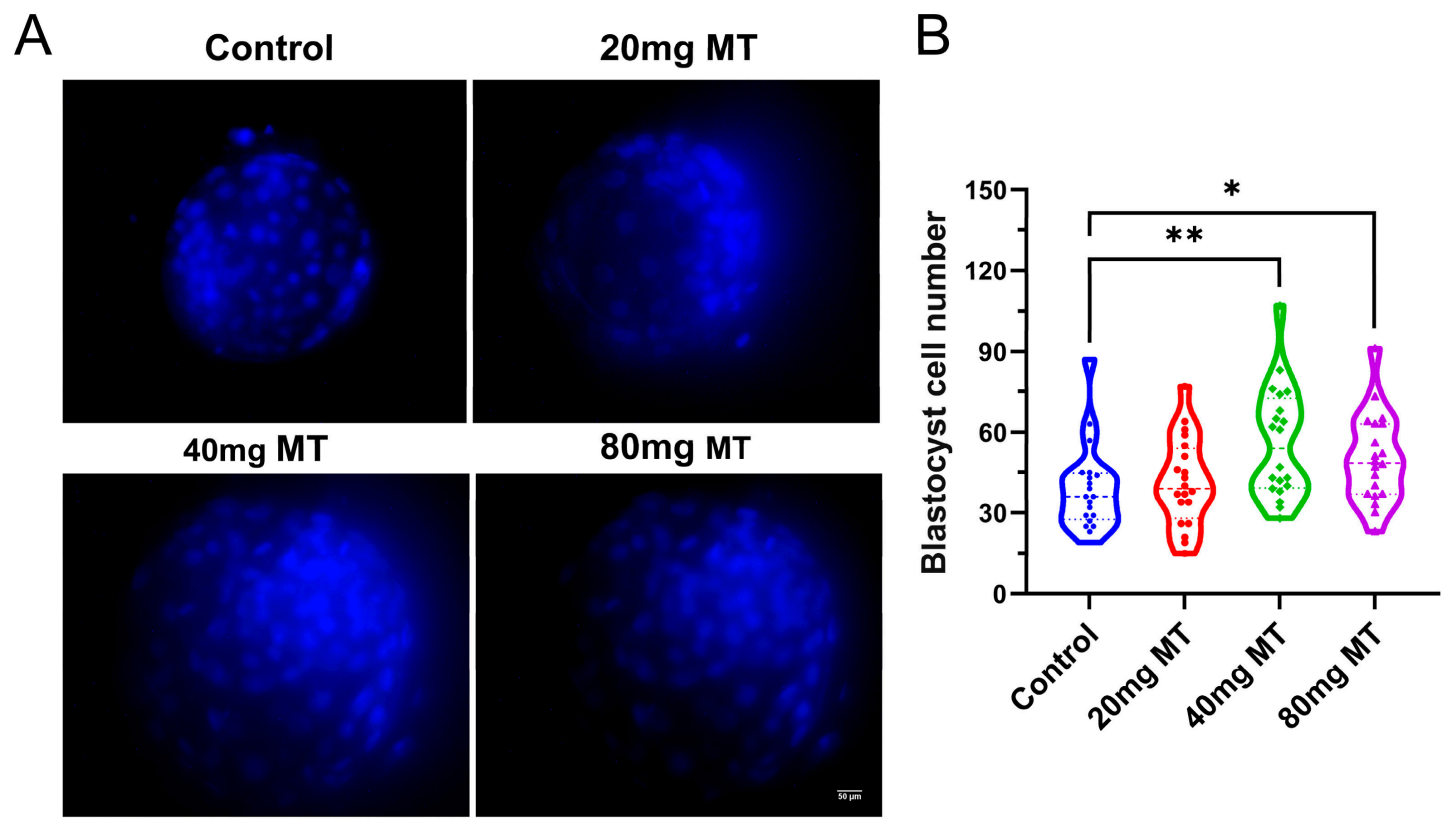
Effects of MT treatment on total blastocyst cell number. (**A**) Representative fluorescence micrographs of Hoechst 33,342-stained blastocysts (Scale bar = 50 μm). (**B**) Violin plot for quantitative analysis of blastocyst total cell number (n = 20 blastocysts per group, pooled from multiple donor ewes within each treatment group; five blastocysts per batch from the first four experimental batches). Data were analyzed using a linear mixed model (LMM) with treatment as a fixed effect and batch as a random effect. * *p* < 0.05, ** *p* < 0.01 vs. control group.

To identify the most favorable MT regimen for the in vitro developmental competence of ovine oocytes, we compared in vitro developmental status and blastocyst formation rates across the four treatment groups. As presented in Table 2, the cleavage rate and total blastocyst rate were significantly greater in the 40 mg MT group relative to the other three cohorts (*p* < 0.05). Quantitative counting of blastocyst cells demonstrated a marked elevation in total cell number per blastocyst in the 40 mg MT group compared with the control group (Figure 2A,B; LMM, *p* = 0.002). The 80 mg MT group also showed significantly higher blastocyst cell numbers than the control group (LMM, *p* = 0.041), although the difference between 40 mg and 80 mg groups was not statistically significant (*p* = 0.275). No significant difference was detected between the 20 mg MT group and the control group (*p* = 0.653). All the results confirmed that 40 mg MT was the most favorable tested regimen for the in vitro developmental competence of ovine oocytes. Therefore, this dose was selected for all subsequent multi-dimensional analyses, including ovarian histomorphology, oocyte functional detection, serum and follicular endocrine profiling, and multi-omics sequencing.

### 3.3. Effects of MT Treatment on Ovarian Morphology, Tissue Architecture and Follicle Population in Ewes

To characterize MT-induced changes in ovarian gross morphology, cortical histology, and follicle distribution, we first assessed ovarian morphometric parameters. To account for the paired nature of bilateral ovaries within each ewe, measurements from the left and right ovaries were averaged for each animal. The 40 mg MT group exhibited significantly greater ovarian length (*p* < 0.05), thickness (*p* < 0.01), weight (*p* < 0.01), and volume (*p* < 0.01) compared with controls, while ovarian width showed an increasing trend that did not reach statistical significance (*p* = 0.057; Table 3).

**Figure 3:**
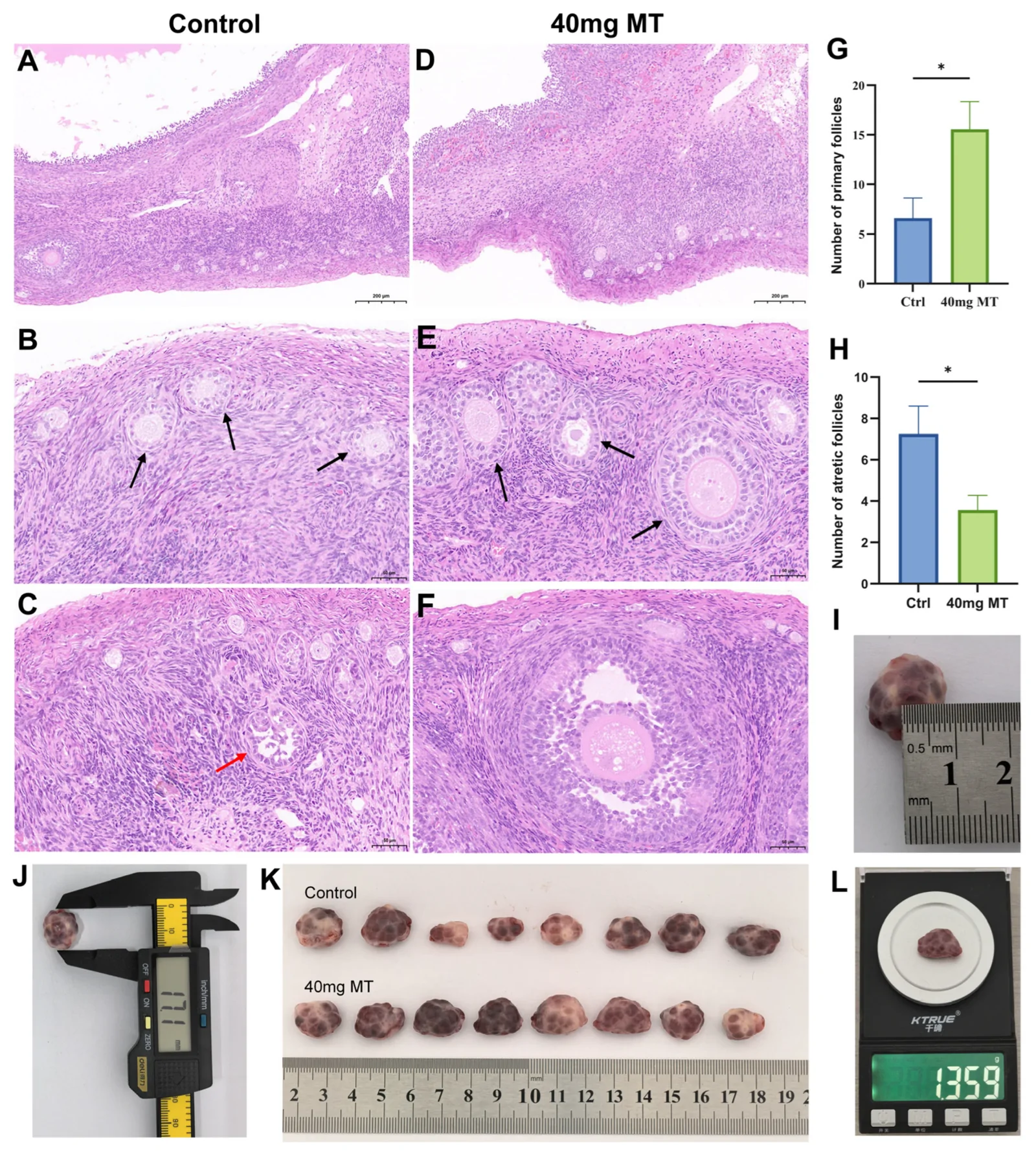
Effects of MT treatment on gross appearance and histological changes in sheep ovaries. (**A**–**F**) Representative low- and high-magnification H&E-stained micrographs of the ovarian cortex. (**G**) Statistical count of primary follicles. (**H**) Statistical count of atretic follicles. (**I**) Schematic diagram for measuring ovarian follicle scale with a ruler. (**J**) Caliper measurement of ovarian length, width and thickness. (**K**) Comparative gross morphology of ovaries. (**L**) Electronic balance weighing of ovarian tissues. Scale bars: (**A**,**D**) = 200 μm; (**B**,**C**,**E**,**F**) = 50 μm. Black arrows denote primary follicles; red arrows mark atretic follicles. Data are presented as mean ± SEM. Ovarian morphometric parameters were based on 13 ewes per group (n = 13). H&E follicle counting was based on six ewes per group in the control group and five ewes in the 40 mg MT group. * *p* < 0.05. The electronic balance brand name is KTRUE (ZiRui, China).

Hematoxylin and eosin (H&E) staining visualized cortical structural integrity (Figure 3A–F). Control ovaries exhibited disorganized cortex and numerous atretic follicles with pyknotic nuclei. Conversely, 40 mg MT preserved intact, ordered ovarian cortex with abundant evenly distributed primary follicles and minimal atresia. Quantitative counting validated elevated primary follicle numbers and reduced atretic follicles in MT-treated ewes (*p* < 0.05, Figure 3G,H). Primordial and secondary follicle counts did not differ significantly between groups (Supplementary File S2).

### 3.4. Effects of Melatonin (MT) Treatment on Key Functional Protein Expression in Ewes

#### 3.4.1. Folliculogenesis-Related Proteins

**Figure 4:**
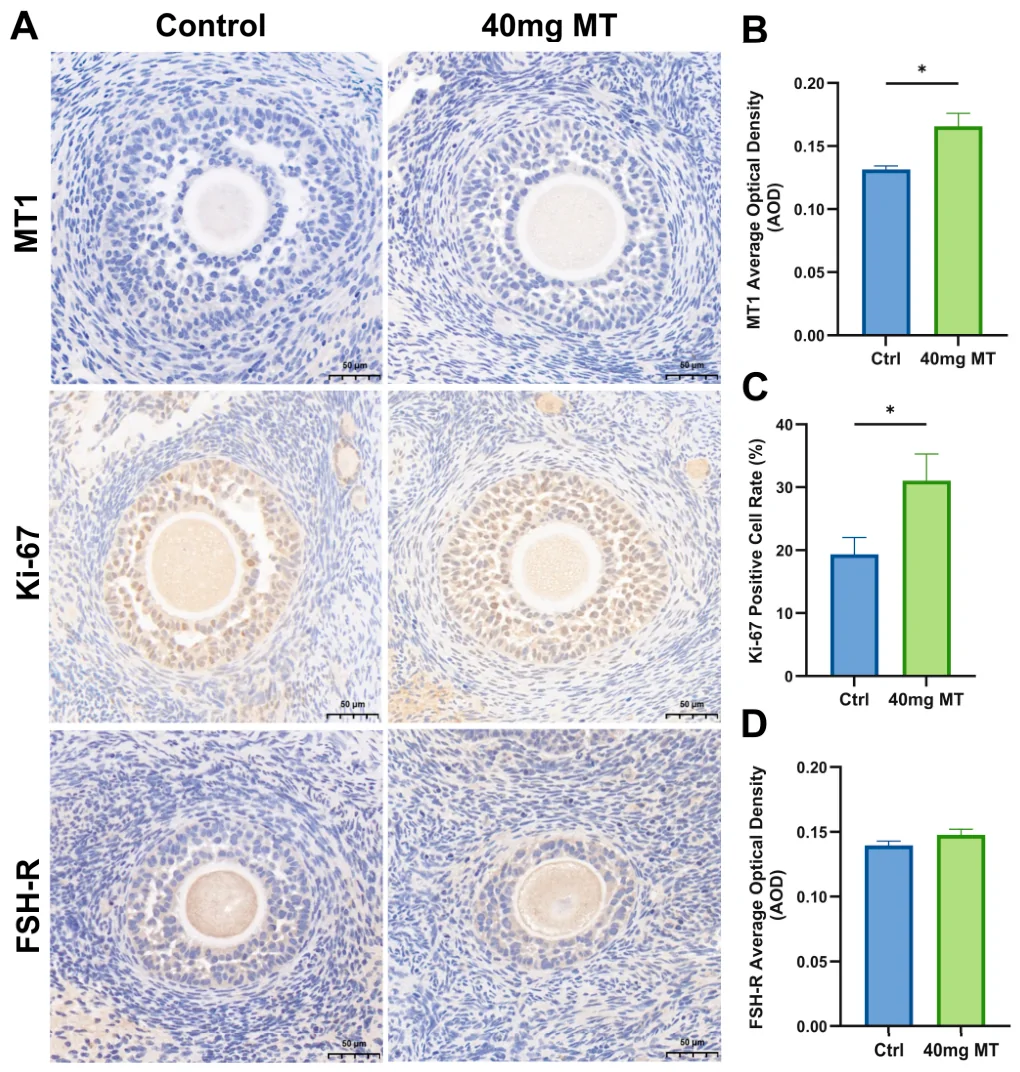
Effects of MT treatment on folliculogenesis-related proteins in ovarian follicles. (**A**) The representative immunohistochemical micrographs (200×) of MT1, Ki-67 and FSH-R in growing follicles. Scale bar: 50 μm; (**B**) The data of quantitative AOD analysis of MT1. (**C**) The statistical data of the proportion of Ki-67-positive granulosa cells. (**D**) AOD quantification of FSH-R. All data are presented as mean ± SEM (n = 6 ewes per group). * *p* < 0.05.

Immunohistochemical localization revealed that MT1, Ki-67 and FSH-R were predominantly expressed in the granulosa cell layer of growing follicles, with only faint positive signals detected within oocytes; no specific staining was observed in the theca or ovarian stroma (Figure 4A). Quantitative analysis demonstrated significantly elevated MT1 average optical density (AOD) in granulosa cells of the 40 mg MT group (*p* < 0.05; Figure 4B). Consistently, the proportion of Ki-67-positive proliferative granulosa cells markedly increased following MT supplementation (*p* < 0.05; Figure 4C). Although FSH-R expression exhibited an upward trend in the MT cohort, the intergroup difference failed to reach statistical significance (*p* > 0.05; Figure 4D; Supplementary File S3).

#### 3.4.2. Apoptosis-Associated Proteins in Granulosa Cells

**Figure 5:**
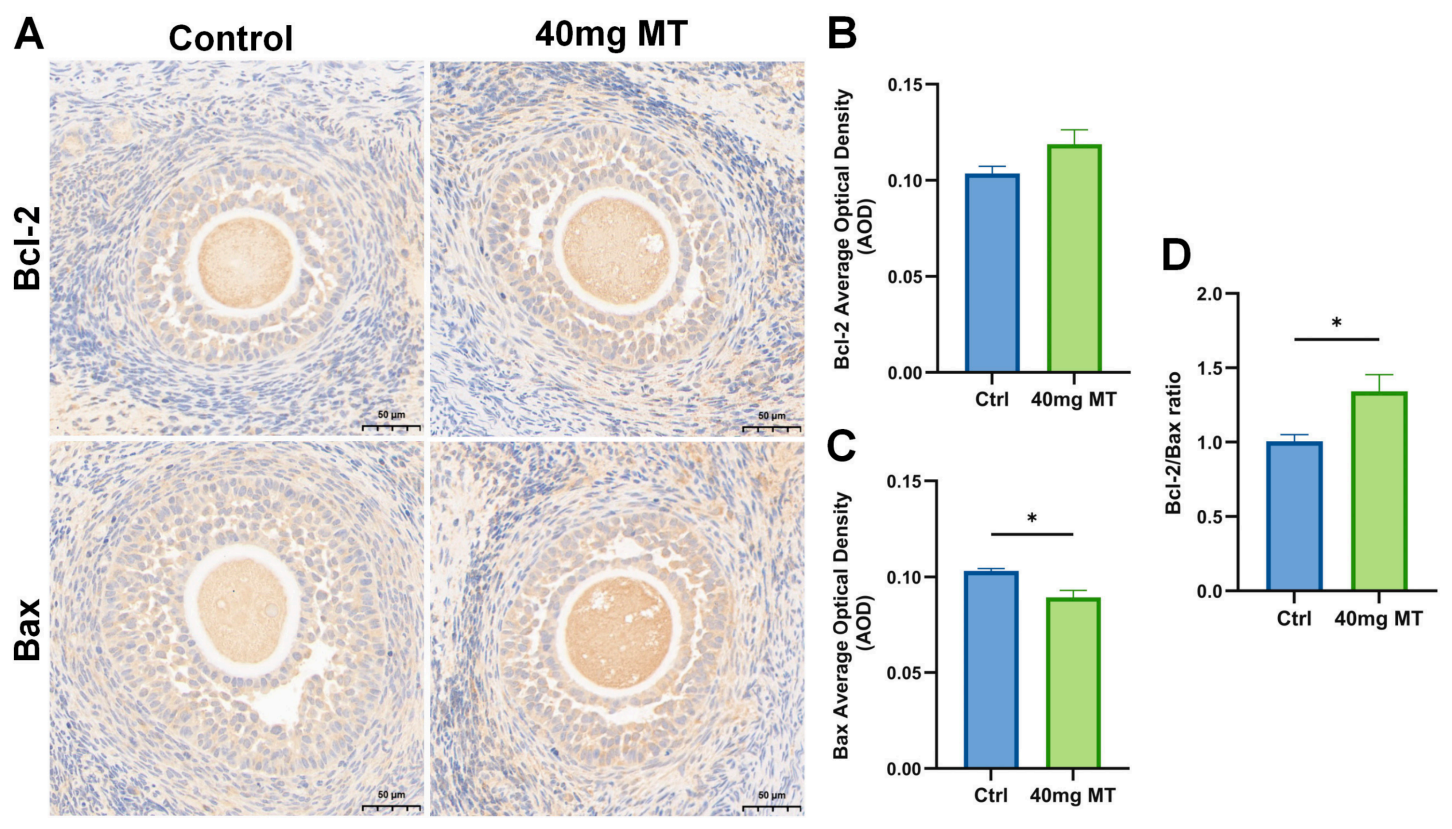
Effects of MT treatment on apoptosis-regulating proteins in granulosa cells. (**A**) The representative immunohistochemical images (200×) of Bcl-2 and Bax in growing follicles. Scale bar: 50 μm. (**B**) AOD analysis of Bcl-2. (**C**) AOD analysis of Bax. (**D**) The Bcl-2/Bax ratio. Data are expressed as mean ± SEM (n = 6 ewes per group). * *p* < 0.05.

The anti-apoptotic Bcl-2 and pro-apoptotic Bax proteins were both mainly localized to granulosa cells (Figure 5A). While Bcl-2 AOD showed a moderate increasing tendency in the 40 mg MT group without statistical significance (*p* > 0.05; Figure 5B), Bax expression was significantly downregulated under MT intervention (*p* < 0.05; Figure 5C). Consequently, the Bcl-2/Bax ratio was remarkably higher in ewes receiving 40 mg MT (*p* < 0.05; Figure 5D; Supplementary File S3).

### 3.5. Effects of MT Treatment on Mitochondrial Function and Redox Homeostasis of Oocytes

**Figure 6:**
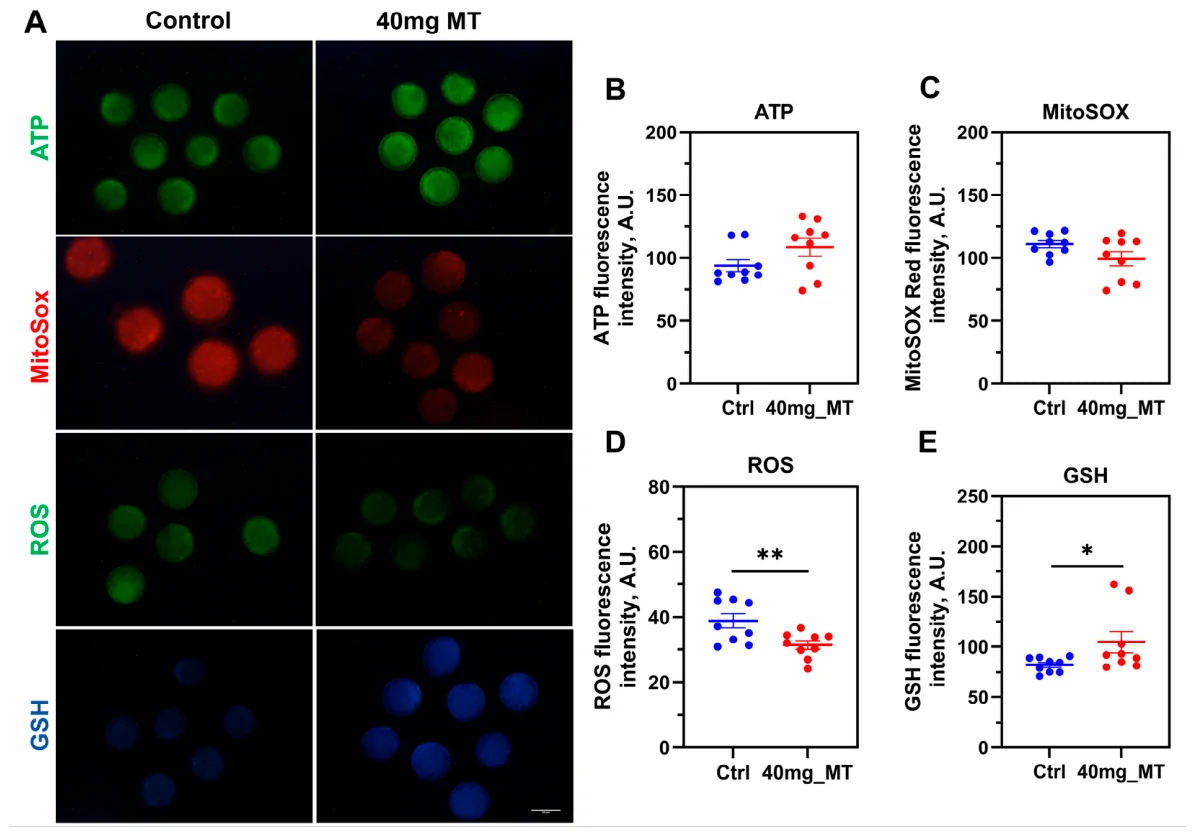
Effects of MT treatment on mitochondrial function and redox homeostasis in ovine oocytes. (**A**) Representative fluorescent micrographs of ATP, MitoSOX, ROS, and GSH signals in oocytes (Scale bar = 100 μm). (**B**–**E**) Quantification of ATP, MitoSOX, ROS, and GSH fluorescence intensities. Each point represents the mean value of multiple oocytes from an individual ewe (n = 9 ewes per group). Data are presented as mean ± SEM. Statistical analysis was performed using a linear mixed model (LMM) with treatment as a fixed effect and ewe as a random effect, using all individual oocyte measurements. * *p* < 0.05, ** *p* < 0.01.

Four specific fluorescent probes were used to quantify the intracellular levels of ATP, GSH, mitochondrial superoxide (mtO_2_^−^), and total ROS in ovine oocytes (Figure 6A). Quantitative fluorescence analysis revealed that intracellular GSH levels were significantly elevated in the 40 mg MT group compared with the control (*p* = 0.038; Figure 6E), whereas intracellular ROS levels were significantly reduced (*p* = 0.003; Figure 6D). ATP levels showed a non-significant increase (*p* = 0.086; Figure 6B) and MitoSOX levels showed a non-significant decrease (*p* = 0.067; Figure 6C), although neither reached statistical significance. These findings indicate that MT treatment was associated with enhanced antioxidant capacity in ovine oocytes, as evidenced by significantly increased GSH and reduced ROS, while ATP and mitochondrial superoxide showed non-significant changes.

### 3.6. Effects of MT Supplementation on Serum Hormone Profiles

**Figure 7:**
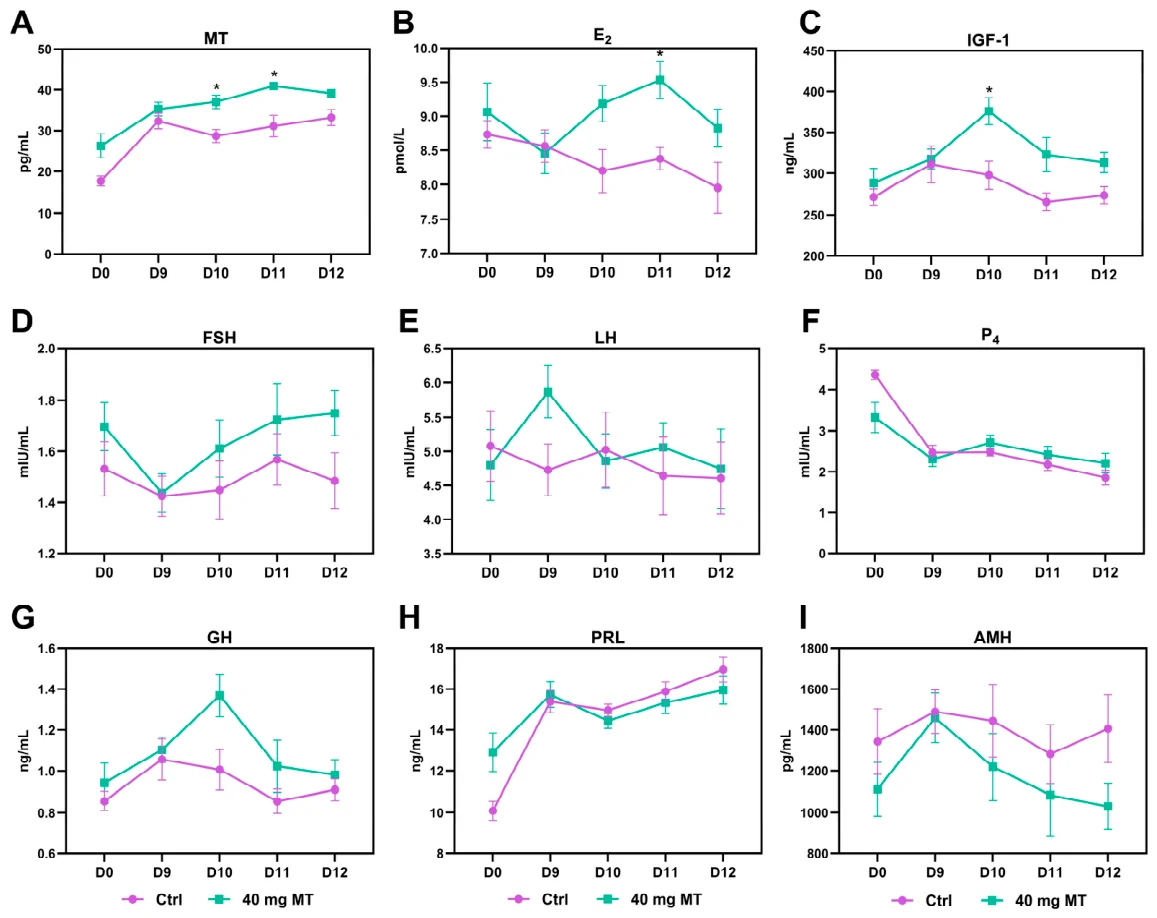
Effects of MT treatment on serum reproductive hormones in ewes. (**A**) MT. (**B**) E_2_. (**C**) IGF-1. (**D**) FSH. (**E**) LH. (**F**) P_4_. (**G**) GH. (**H**) PRL. (**I**) AMH. Blood sampling was conducted at Day 0 (baseline before CIDR placement) and each morning before drug injection from Day 9 to Day 12. All data are expressed as mean ± SEM (n = 7). * *p* < 0.05.

Blood samples were collected via jugular venipuncture at baseline (Day 0, prior to CIDR insertion) and each morning before MT treatment from Day 9 to Day 12 to dynamically detect serum reproductive hormone concentrations. No intergroup differences were observed for any hormonal indicators at the Day 0 baseline timepoint. Two-way repeated-measures ANOVA showed significant treatment effects for MT, E2, and IGF-1 (*p* < 0.05), but not for FSH, LH, P4, GH, PRL, or AMH (*p* > 0.05). Significant time effects were observed for MT, IGF-1, P4, GH, and PRL (*p* < 0.05), but not for E2, FSH, LH, or AMH (*p* > 0.05). Notably, significant treatment × time interactions were observed for P4 (*p* = 0.0037) and PRL (*p* = 0.0103), indicating that the temporal patterns of these hormones differed between groups, even though the overall treatment effect was not significant. Subsequent Šidák-adjusted pairwise comparisons did not reveal significant between-group differences at any individual time point for either P4 or PRL (all adjusted *p* > 0.05). No significant treatment × time interactions were found for the other hormones (*p* > 0.05; Table 4). Circulating endogenous MT levels were significantly higher in the 40 mg MT group on Day 10 and Day 11 (*p* < 0.05, Figure 7A). In addition, E_2_ and insulin-like growth factor-1 (IGF-1) concentrations were significantly elevated on Day 11 and Day 10, respectively (*p* < 0.05, Figure 7B,C). Although the 40 mg MT group exhibited rising trends in FSH, LH, and GH, these shifts failed to reach statistical significance at any individual time point (*p* > 0.05, Figure 7D,E,G). No remarkable between-group disparities in AMH concentrations were detected throughout the whole sampling period (*p* > 0.05, Figure 7I).

### 3.7. Effects of Melatonin (MT) Intervention on Physiological and Biochemical Indices of Follicular Fluid

**Figure 8:**
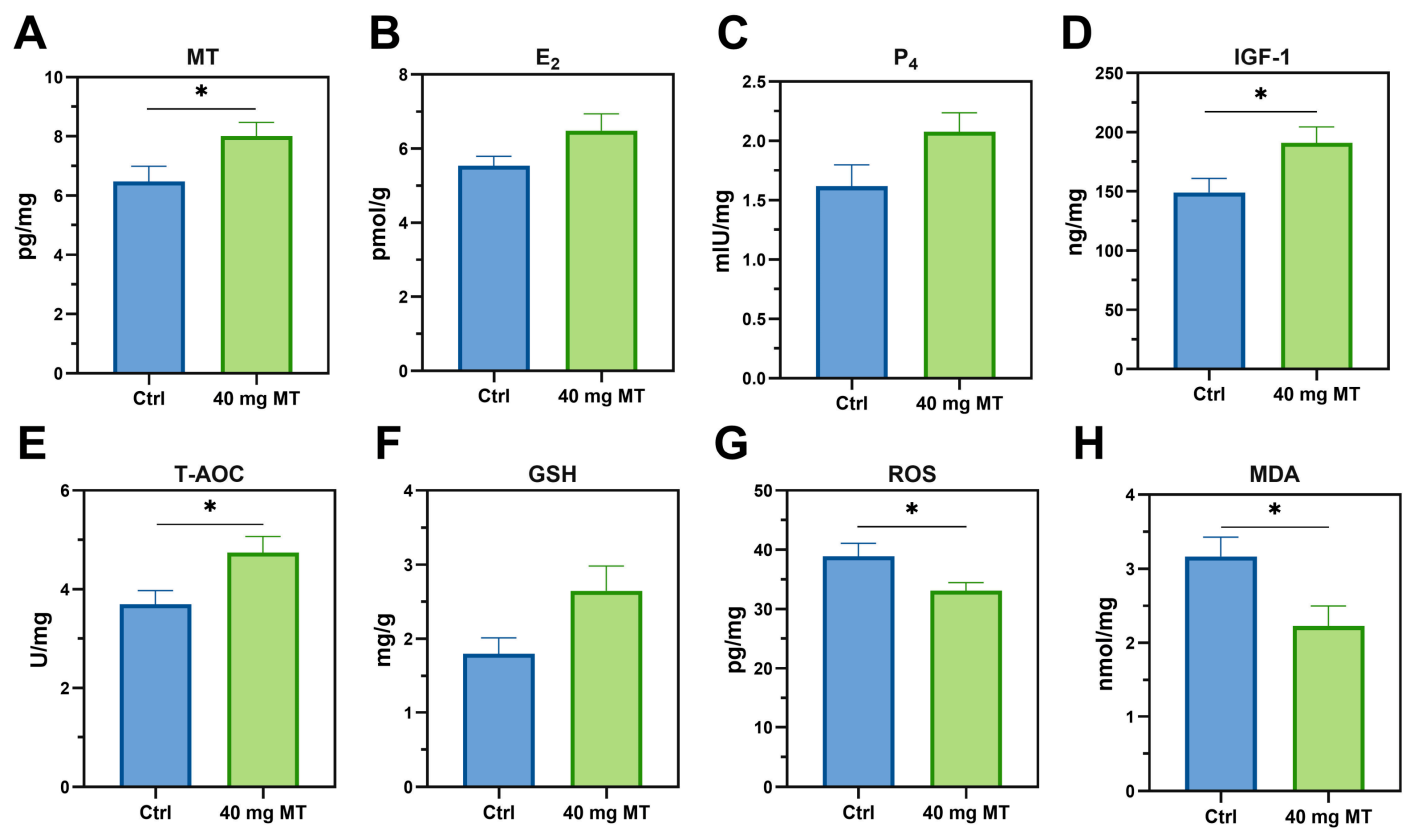
Physiological and biochemical indicators of follicular fluid after MT treatment. (**A**) Follicular MT concentration. (**B**) E_2_ level. (**C**) P_4_ concentration. (**D**) IGF-1 level. (**E**) T-AOC. (**F**) GSH concentration. (**G**) ROS level. (**H**) MDA content. All data are presented as mean ± SEM. (Ctrl: n = 6; 40 mg MT: n = 8). * *p* < 0.05.

Key hormones, growth factors and oxidative stress markers within follicular fluid were quantified (Figure 8). The 40 mg MT group displayed significantly elevated levels of follicular MT, IGF-1 and T-AOC (*p* < 0.05, Figure 8A,D,E). Meanwhile, follicular ROS and MDA contents were markedly reduced in MT-treated ewes (*p* < 0.05, Figure 8G,H). No statistically distinguishable intergroup variations were found in E_2_, P_4_ and GSH concentrations (*p* > 0.05, Figure 8B,C,F).

### 3.8. Impacts of MT Treatment on In Vivo Developmental Outcomes Following In Vitro Blastocyst Transfer

In vitro-derived blastocysts generated through IVM/IVF/IVC procedures were transferred to estrus-synchronized recipient ewes, and pregnancy rate and lambing success among pregnant recipients were assessed to evaluate the effect of MT treatment on embryonic developmental competence (Table 5). Numerically, the pregnancy rate reached 55.6% (15/27) in the 40 mg MT group versus 46.2% (12/26) in the control group, representing an absolute increase of 9.4 percentage points. Compared with the control group, the 40 mg MT group had a relative risk (RR) of pregnancy of 1.20 (95% CI: 0.70–2.06); however, this difference was not statistically significant (two-sided Fisher’s exact test, *p* = 0.587). Among pregnant recipients, lambing success was 93.3% (14/15) in the 40 mg MT group and 83.3% (10/12) in the control group, corresponding to an absolute rise of 10.0 percentage points. The RR for successful lambing was 1.12 (95% CI: 0.84–1.49), and the between-group difference was not statistically significant (*p* = 0.569).

### 3.9. Effects of MT on Ovine Oocyte Transcriptome and qRT-PCR Technical Confirmation

**Figure 9:**
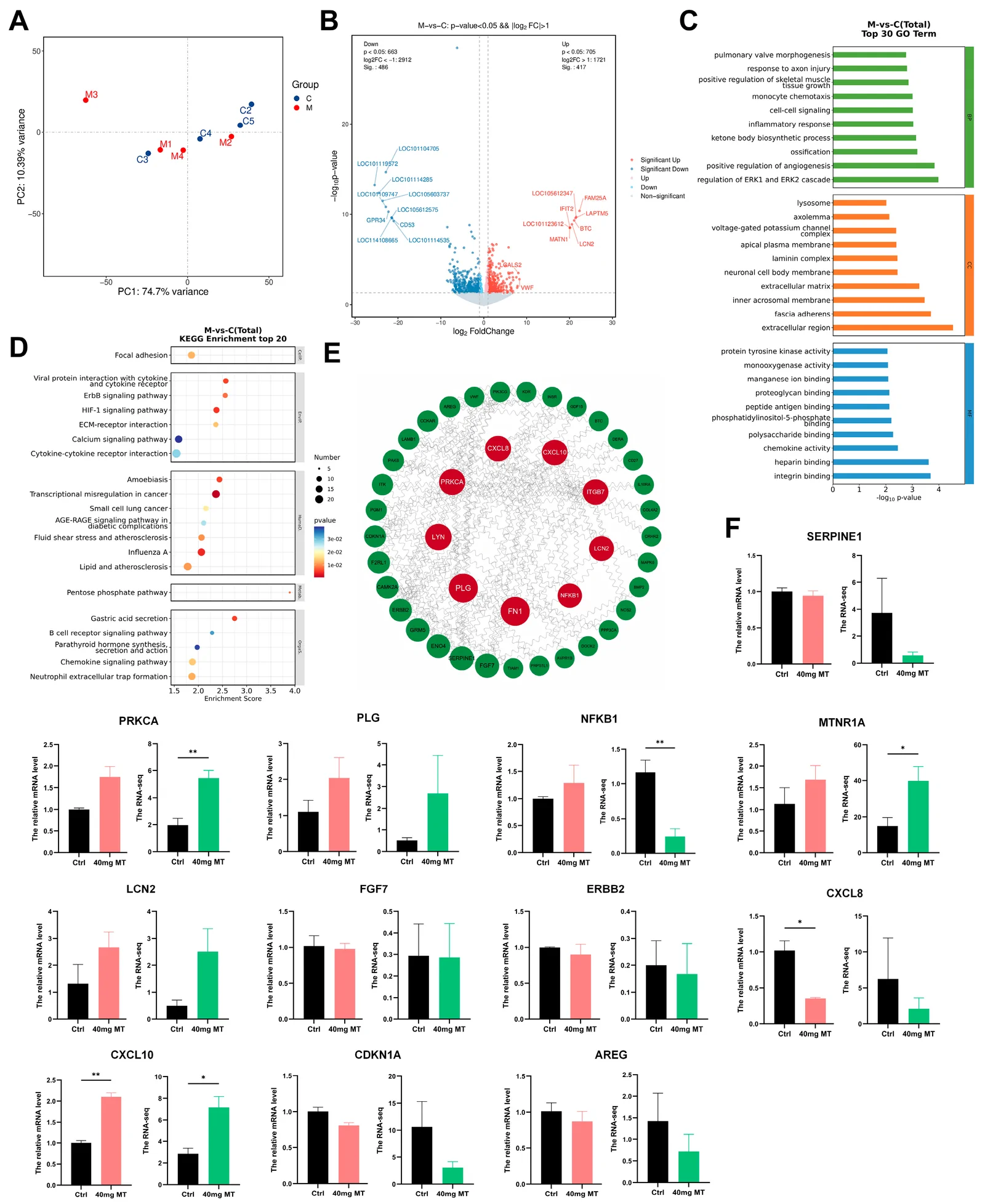
Transcriptomic profiling and qRT-PCR technical confirmation of oocytes from control and 40 mg MT-treated ewes. (**A**) Principal component analysis (PCA) showing distinct transcriptional signatures and intra-group clustering of control (**C**) and 40 mg MT (M) samples. (**B**) Volcano plot of differentially expressed genes (DEGs), with red dots representing significantly upregulated genes and blue dots representing significantly downregulated genes. (**C**) Top 30 GO term enrichments of DEGs. (**D**) Top 20 KEGG pathway enrichments of DEGs, where dot size corresponds to the number of enriched genes and dot color correlates with *p*-value. (**E**) Protein–protein interaction (PPI) network of DEGs and identification of core hub genes. (**F**) qRT-PCR technical confirmation of candidate DEGs. * *p* < 0.05, ** *p* < 0.01.

Principal component analysis (PCA) revealed obvious separation between control (C) and 40 mg MT-treated (M) groups, with well-clustered replicates within each group (Figure 9A). A total of 903 differentially expressed genes (DEGs) were identified, including 417 upregulated and 486 downregulated genes (Figure 9B).

GO and KEGG enrichment analyses indicated that DEGs were predominantly enriched in biological process regulation, signal transduction, immune response, cell adhesion, and developmental pathways, with significant enrichment in calcium signaling, ErbB, HIF-1 and chemokine pathways (Figure 9C,D; Supplementary File S4). A PPI network was constructed based on the 76 DEGs, and hub genes were identified (Figure 9E). FN1, PLG and PRKCA served as core nodes, and multiple high-connectivity hub genes, including NFKB1, were screened out. Eleven candidate key genes were selected from these hub genes, and MTNR1A was selected because it was a DEG encoding the melatonin receptor, although it was not connected in the STRING network. These 12 genes were used for qRT-PCR technical confirmation, and their expression trends were consistent with the RNA-seq data (Figure 9F).

### 3.10. Effects of MT on the Metabolome of Follicular Fluid

**Figure 10:**
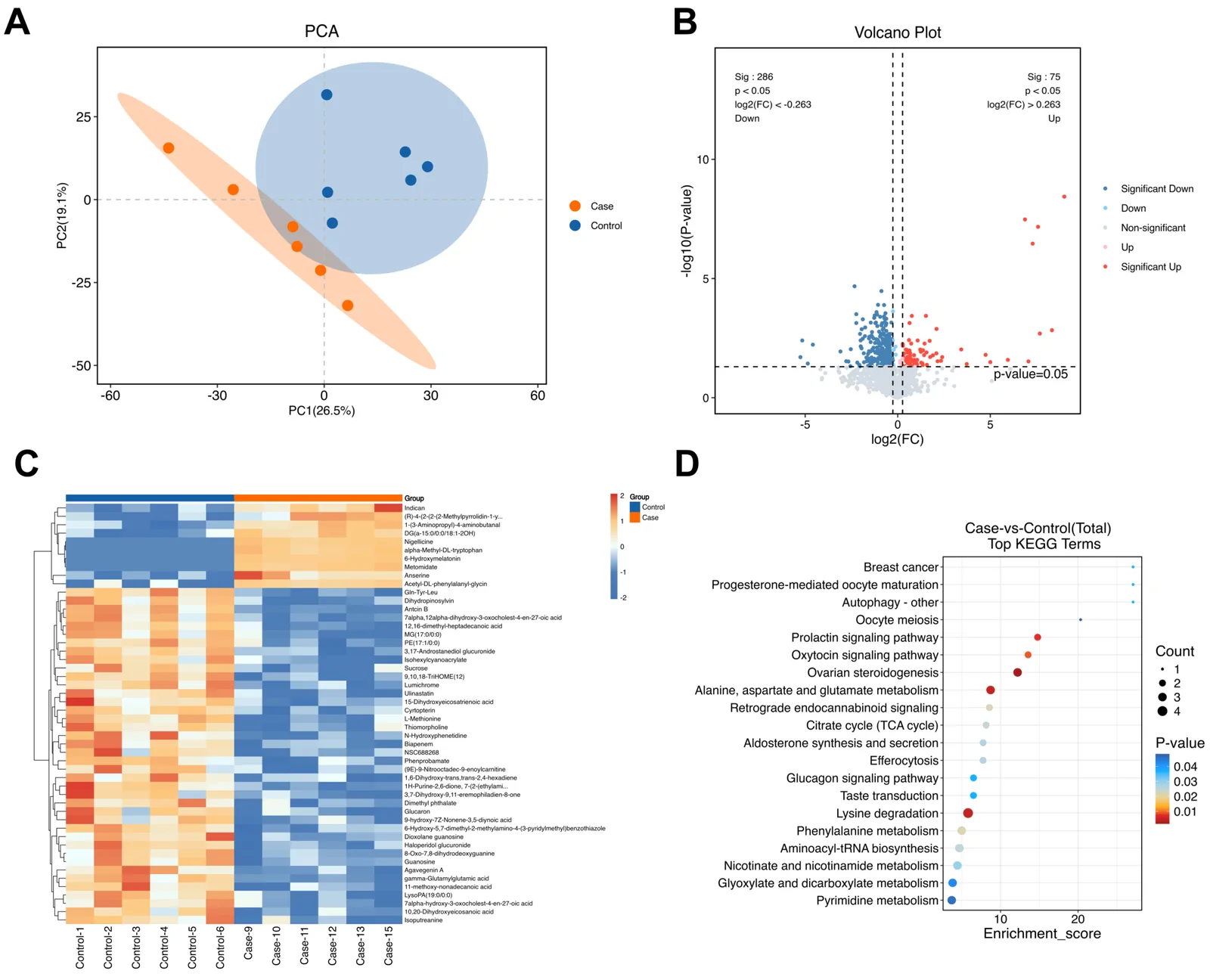
Untargeted LC-MS/MS metabolomic profiling of follicular fluid from control and 40 mg MT-treated ewes. (**A**) PCA of follicular fluid samples. (**B**) Volcano plot of differential metabolites, with red dots for significantly upregulated metabolites and blue dots for significantly downregulated metabolites. (**C**) Clustering heatmap of the top 50 differential metabolites. (**D**) KEGG enrichment analysis of differential metabolites. Dot size corresponds to the number of enriched metabolites, and dot color correlates with the *p*-value.

PCA exhibited distinct segregation between the control group and the 40 mg MT group, with tight intra-group clustering of all samples (Figure 10A). A total of 361 differential metabolites were characterized, among which 75 were upregulated and 286 were downregulated (Figure 10B, Supplementary File S5). The clustering heatmap of the top 50 differential metabolites further confirmed divergent metabolic profiles between the two cohorts (Figure 10C).

KEGG enrichment analysis demonstrated that these differential metabolites were significantly enriched in pathways including ovarian steroidogenesis, tricarboxylic acid (TCA) cycle, amino acid metabolism, progesterone-mediated oocyte maturation and oocyte meiosis (Figure 10D). Transcriptomic and metabolomic datasets originated from independent cohorts of experimental ewes without matched individual samples, so only comparative trend analysis at the population level was feasible, rather than precise multi-omics association analysis for single specimens.

## 4. Discussion

This study established an in vivo model of FSH-synchronized superovulated adult Hu sheep with gradient subcutaneous MT administration, and systematically compared the effects of four MT dose regimens in ruminants. Treatment with 40 mg MT significantly improved follicle quantity and oocyte in vitro developmental capacity, whereas the favorable responses observed at 40 mg were not maintained at 80 mg. This dose-dependent response pattern may be consistent with the MT1/MT2 receptor-balance hypothesis [29,30], but this interpretation was not directly tested in the present study. It should be noted that the MT1/MT2 receptor-balance interpretation is a post hoc mechanistic hypothesis generated from the observed phenotypic differences and the previous literature, rather than an a priori hypothesis directly tested in this study. Based on previous in vitro studies, MT is thought to preferentially bind MT1 receptors at physiological concentrations, initiating cAMP/PKA-dependent antioxidant and anti-apoptotic programs. Supraphysiological MT concentrations may disrupt this balance and overactivate MT2 pathways, potentially disturbing lipid metabolism and promoting lipid peroxide accumulation. These receptor-mediated mechanisms were not directly examined in the present study. Although this lipid-disrupting effect has been reported in mouse follicular cell models [31], it remains hypothetical for ovine granulosa cells and was not tested in this study. This finding is highly consistent with the report of Adriaens et al. [31], whose gradient experiment ranging from 0.1 nM to 2 mM MT observed cellular toxicity and suppressed oocyte maturation at 2 mM, yet elevated progesterone secretion at 100 μM, indicating that dose-dependent responses to MT appear to be conserved across species, although the underlying molecular mechanism has not been proven to be the same in both species. The favorable responses observed at 40 mg were not maintained at 80 mg, which is broadly consistent with the receptor-mediated dose–response pattern reported in the literature, although contributions from nonspecific free radical scavenging or other off-target effects cannot be excluded, as receptor signaling was not directly measured. In vitro culture systems lack the buffering of endogenous hormonal negative feedback and maintain constant exogenous MT concentrations; the in vivo physiological response, including the decline in beneficial effects at higher doses, might not be fully reproduced in vitro. In adult Hu sheep, sustained circulating E_2_ and IGF-1 participate in homeostatic regulation, which may contribute to the decline in beneficial effects at higher doses. Such in vivo–in vitro discrepancies highlight the unique value of multi-gradient in vivo models for restoring authentic physiological responses. Most existing studies employed single fixed concentrations in vitro or in rodent models, failing to recapitulate the complete hypothalamic–pituitary–ovarian endocrine milieu [32,33,34]; our multi-gradient in vivo evaluation system thus provides a useful methodological reference for analyzing MT’s dose-dependent regulatory characteristics. However, this study only confirmed that the favorable responses observed at 40 mg were not observed at 80 mg at the macroscopic phenotypic level, without further detection of follicular lipid peroxides and oocyte mitochondrial membrane potential, so the specific molecular mechanisms underlying the decline in beneficial effects at higher doses remain to be verified in subsequent experiments.

Ovarian histological and immunohistochemical results further provided insights into the cytological mechanisms underlying MT’s protective effects on follicle reserve. Treatment with 40 mg MT maintained intact ovarian cortical structure, upregulated granulosa cell proliferation marker Ki-67, increased the Bcl-2/Bax ratio, and reduced the number of atretic follicles, whereas primordial follicles showed no intergroup differences. The above phenotypes are consistent with the results of in vitro granulosa cell tests in chickens [35], mice [36], sheep [15] and cattle [37], forming mutual verification between in vivo histological and in vitro molecular evidence. The intrafollicular naturally hypoxic environment continuously induces excessive ROS accumulation, and oxidative stress acts as a core trigger for granulosa cell programmed death and follicular atresia [35,38]. Immunohistochemical localization revealed specific high expression of MT1 in granulosa cell layers, indicating that circulating MT can directly target granulosa cell membrane receptors to exert its functions. Wang et al. [39] proved that the anti-apoptotic effects of low-concentration MT can be completely blocked by combined administration of Luzindole and 4P-PDOT, while high-dose MT functions partially independently of the two receptors. These findings suggest that MT1 signaling may be involved in the protective effects observed at 40 mg, but receptor-blocking or gene-interference experiments were not performed, so a causal role of MT1 cannot be established. Other molecular pathways may contribute to the decline in beneficial effects at 80 mg, but this also remains to be verified. Nevertheless, only semi-quantitative detection of tissue proteins was performed in the current study; follow-up in vitro experiments using ovine primary granulosa cells with receptor blocking or gene interference are required to verify the inferred relevant pathways.

Mitochondrial function and intracellular redox homeostasis constitute the core determinants of oocyte cytoplasmic maturation potential. After 40 mg MT intervention, intracellular GSH content was significantly increased and ROS level was significantly reduced, accompanied by non-significant increases in ATP and non-significant decreases in mitochondrial superoxide levels. In vitro studies on pigs and cattle have confirmed that mitochondria are the primary sites of endogenous MT synthesis in oocytes, and oocytes express AANAT and ASMT synthases. Endogenous MT stabilizes mitochondrial membrane potential and alleviates oxidative stress-induced spindle damage [40,41]. Compared with static in vitro culture systems, sustained MT enrichment in follicular fluid via in vivo subcutaneous injection may be associated with changes in oocyte oxidative phosphorylation efficiency, although ATP levels did not reach statistical significance in this study. Follicles display stage-specific metabolic characteristics [42,43,44]: small and medium follicles maintain basal antioxidant capacity, while dominant preovulatory follicles physiologically accumulate ROS before ovulation. MT may help narrow the gap of oxidative and energy supply among follicles and may be associated with improved oocyte cytoplasmic developmental competence [45]. Consistent with our oocyte mitochondrial phenotypic results, previous research on bovine granulosa cells also confirmed that MT promotes mitophagy and relieves oxidative stress-induced apoptosis through the SIRT1/FoxO1 signaling pathway [14].

The changes in mitochondrial oxidative and energy homeostasis observed at the cellular level were further explored via combined oocyte transcriptomics and follicular fluid metabolomics, and the findings should be considered hypothesis-generating. Transcriptomic analysis showed that differentially expressed genes were significantly enriched in calcium signaling, ErbB and HIF-1 pathways; RT-qPCR verification demonstrated consistent expression trends of FGF7, AREG, MTNR1A and other key genes with sequencing data. Disordered calcium signaling disrupts oocyte spindle assembly [46] and the ErbB pathway regulates cumulus cell gap junction communication and intercellular nutrient transport [47]. Metabolomic profiling of follicular fluid revealed that differential metabolites were mainly enriched in the TCA cycle and ovarian steroidogenesis pathways. Studies on dairy cows and pigs have proven that excess free fatty acids trigger lipotoxicity and mitochondrial damage [48,49]. The enrichment of the TCA cycle was observed together with a non-significant increase in oocyte ATP levels; therefore, this association remains hypothetical and requires further validation. Although oocyte transcriptomic and follicular fluid metabolomic samples were collected from independent sheep individuals without paired single specimens, the concordance between the two independent datasets provides convergent biological evidence, although it cannot exclude batch effects or establish individual associations. The consistent enrichment of core energy-redox pathways suggests that MT may be associated with remodeling of the intrafollicular energy-redox microenvironment, which could potentially influence molecular cascades governing folliculogenesis and oocyte cytoplasmic maturation. Restricted by unpaired samples for the two omics platforms, such correlation only reflects population-level trends, and paired multi-omics trials are needed to further confirm causal relationships.

The above changes in mitochondrial redox status and molecular network remodeling rely on the material and signal support of the intrafollicular microenvironment, and this microenvironment is directly regulated by the systemic hypothalamic-pituitary-ovarian endocrine axis [50,51]. In the 40 mg MT group, serum MT, E_2_ and IGF-1 were significantly upregulated, and follicular fluid MT, IGF-1 and T-AOC also increased synchronously, accompanied by decreased follicular fluid ROS and MDA contents. Notably, Picinato et al. [52] reported in rat pancreatic islets that MT activates IGF-1 receptor tyrosine phosphorylation through MT1 receptors to further mediate downstream PI3K/AKT and MEK/ERKs signaling cascades. This mechanistic evidence suggests that MT may enhance IGF-1 signaling efficiency via MT1-dependent mechanisms to strengthen intrafollicular IGF-1 signaling. Existing studies have verified that IGF-1 activates the PI3K/Akt pathway to simultaneously promote granulosa cell proliferation and boost antioxidant capacity [53,54], and ovine-specific research proved that MT alleviates the inhibitory impacts of high estradiol on granulosa cells [55,56]. Integrating phenotypic data and the published literature, we infer that exogenous MT exerts synergistic antioxidant effects through two approaches: raising circulating and intrafollicular IGF-1 concentrations, and amplifying IGF-1 signaling transduction through MT1 receptors. FSH, LH, and AMH only presented upward trends without statistical significance at individual time points, which is attributed to endogenous hormonal homeostatic compensation in adult sheep [57]. It is worth noting that P4 and PRL exhibited significant treatment × time interactions, suggesting that MT treatment may alter the temporal dynamics of these hormones even without significant between-group differences at individual time points. Further studies are needed to understand the physiological significance of these temporal differences. Short-term superovulation cannot permanently alter basal hormone secretion levels, which aligns with conclusions from previous in vivo MT trials in sheep [7,58].

The protective phenotypes observed at molecular and cellular levels were supported by in vitro embryonic development outcomes. In vivo embryo transfer pregnancy and lambing rates were numerically higher in the 40 mg group but did not reach statistical significance. Under IVF culture conditions, the cleavage rate, total blastocyst rate and single blastocyst cell number of the 40 mg MT group were all significantly superior to the control group, which confirmed that MT effectively improves oocyte cytoplasmic maturation quality. The results from in vivo embryo transfer showed numerically higher pregnancy and lambing rates in melatonin-treated ewes compared with controls, but these differences did not reach statistical significance. Nevertheless, these numerical differences provide a rationale for large-scale future investigations by our group and others. This outcome partly diverges from our previous Small-Tailed Han sheep study, in which MT implants were given to both donors and recipients and significantly improved pregnancy and lambing [19]. Such divergence most likely stems from different administration regimens: implants provided long-term continuous MT exposure for both donor follicles and the recipient uterine environment, whereas our current intervention consisted of intermittent MT injections limited exclusively to donor ewes during the follicular superovulation period, with no MT treatment for recipients. This is consistent with our sika-deer observation that MT biological effects are strongly dependent on administration mode and experimental context [20]. Three reasons may explain this inconsistency: First, follicular maturation merely constitutes a prerequisite for gestation, and livestock reproduction requires coordinated function of the ovary, uterus and corpus luteum; MT intervention in this study was limited to the follicular phase during superovulation, without modulating endometrial receptivity-related pathways. Second, adult sheep possess complete endogenous antioxidant and hormonal homeostatic compensation systems that narrow intergroup differences in embryonic quality; in vitro culture removes complex maternal endocrine feedback and magnifies the beneficial effects of MT. Third, the limited number of embryo transfer recipients reduced the statistical power of Fisher’s exact test; such small numerical improvements could not reach the significance threshold. This observation is consistent with the sheep follicular-phase MT intervention data reported by Abecia et al. [18]. Conflicting conclusions exist among published ovine MT trials: Yesilkaya et al. [58] subcutaneously implanted 18 mg MT before superovulation and detected significantly lower corpus luteum counts and recovered oocytes, consistent with the decline in beneficial effects at 80 mg MT in our research. In contrast, Song et al. [25] found that subcutaneous injection of 5 mg MT markedly increased superovulated embryo yields and recipient pregnancy rates. Divergent dosages and sample sizes account for such inconsistent results, yet both studies confirm that MT increases the total number of recoverable gametes. From the perspective of large-scale elite sheep multiplication, even without statistically significant reproductive indices, 40 mg MT can greatly increase the output of follicles, high-quality oocytes and transferable blastocysts. The same number of donor ewes can generate more embryos for transfer, which may help to shorten elite breeding cycles and reduce production costs, although formal cost-effectiveness analyses were not performed.

**Figure 11:**
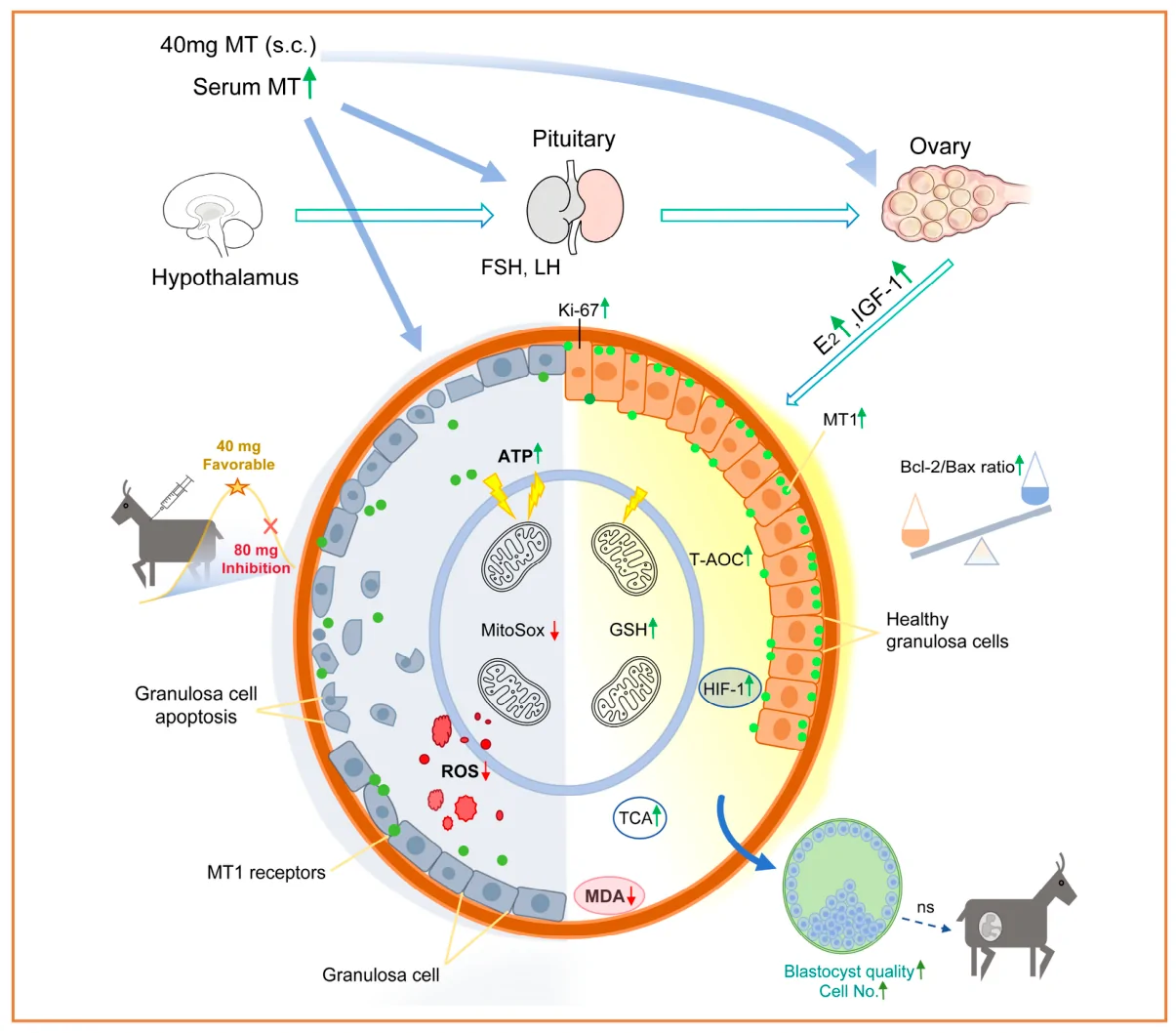
The proposed model summarizing the potential protective effects of MT on ovine folliculogenesis and oocyte maturation. Red arrows indicate downregulation, green arrows indicate upregulation.

Compared with previous studies adopting single-dose in vitro culture or rodent models, the present study constructed a complete 20, 40, 80 mg in vivo gradient system in adult Hu sheep, and clarified the dose-related regulatory characteristics of MT under intact hypothalamic-pituitary endocrine background via cross-validation of two independent omics datasets. This study has several inherent limitations: First, the MT1-mediated oxidative and anti-apoptotic regulatory cascade was only deduced based on in vivo phenotypes and the published cellular literature; follow-up trials isolating ovine primary granulosa cells with MT1/MT2 receptor antagonists and siRNA knockdown are required to confirm direct molecular causality. Second, oocyte transcriptomic and follicular fluid metabolomic data originated from separate sheep cohorts without matched individual specimens; only population-level pathway comparisons can be conducted instead of precise single-sample multi-omics association analysis. Third, the limited cohort of embryo transfer recipients weakened statistical power; MT treatment was restricted to the follicular phase without intervening pathways governing endometrial receptivity and corpus luteum function, so merely optimizing oocyte redox balance cannot offset the physiological compensation of sheep endogenous reproductive systems. In addition, because oocytes and embryos were pooled within each treatment group, individual donor-to-recipient mapping was not available, and donor-level clustering could not be accounted for in the analysis of pregnancy and lambing outcomes. Because donor-to-recipient mapping was not retained, Fisher’s exact tests cannot account for potential clustering by donor ewe, which may have influenced the interpretation of these reproductive outcomes. This may underestimate the dependency structure and should be addressed in future studies by tracking individual donor-embryo-recipient relationships. Although the blastocyst total cell number analysis accounted for batch-level clustering using a linear mixed model, it did not account for donor-level clustering because individual donor origin was not recorded. Fourth, the favorable responses observed at 40 mg were not maintained at 80 mg, and no molecular detection of follicular lipid metabolism or oocyte mitochondrial membrane potential was performed, so the specific mechanisms underlying the decline in beneficial effects at higher doses remain unclear. To address these limitations, follow-up targeted experiments can be carried out: isolate ovine primary cumulus granulosa cells and utilize Luzindole and 4P-PDOT receptor inhibitors to verify MT downstream signaling; collect paired oocyte-follicular fluid samples for integrated multi-omics analysis; expand the sample size of embryo transfer recipients; perform functional verification on hub genes such as HIF1A and PRKCA screened from transcriptome and metabolome data. Future work should evaluate whether simultaneous MT treatment of donors and recipients, similar to our former implant-based strategy, could further amplify the reproductive benefits observed in this Hu-sheep injection model. From the perspective of standardized FSH superovulation and in vivo ovum pick-up in sheep breeding, simultaneous subcutaneous administration of 40 mg MT alongside FSH can stably maintain ovarian redox balance, reduce follicular atresia and elevate yields of high-quality gametes and transferable blastocysts. Based on multi-dimensional in vivo data, this work describes candidate molecular associations between redox homeostasis and MT-related effects on follicular development and oocyte maturation, but does not establish a complete molecular network or causal relationships. This ruminant-specific gradient in vivo multi-dimensional evaluation framework provides systematic in vivo experimental support for basic research on ovarian oxidative injury, oocyte mitochondrial metabolism, and the construction of in vitro embryo production systems for mutton sheep. Collectively, these findings support a model in which MT-associated redox remodeling may contribute to the regulation of ovine folliculogenesis and oocyte quality, with MT1 signaling representing a plausible upstream mechanism requiring functional validation. The overall regulatory model is summarized in Figure 11.

## 5. Conclusions

From the perspective of standardized FSH superovulation and in vivo ovum pick-up in sheep breeding, subcutaneous administration of the 40 mg MT regimen alongside FSH was associated with changes in ovarian redox status, reduced follicular atresia, and elevated yields of high-quality gametes and transferable blastocysts in superovulated ewes. Pregnancy and lambing rates after in vivo embryo transfer were numerically higher but did not reach statistical significance. The favorable responses observed with the 40 mg regimen were not maintained at the 80 mg dose. To our knowledge, this is the first study to compare multiple MT dose regimens in ewes. At the systemic endocrine level, the 40 mg MT regimen was associated with significant increases in serum MT, E2, and IGF-1. At the cellular and molecular levels, MT treatment was associated with changes in ovine folliculogenesis and oocyte quality, and MT1-related signaling was identified as a candidate pathway, together with altered redox status and candidate changes in energy metabolism. Integrative omics analyses identified HIF-1 signaling and the TCA cycle as candidate pathways associated with follicular energy–redox remodeling. However, this study was not designed to establish causal mechanisms or to define an optimal dose, and the proposed pathways remain candidates that require functional validation. These observations are summarized in Figure 11.

## Figures and Tables

**Table 1:** Primer information.

Gene	Accession No.	Primer Sequences (5′-3′)		Anneal Temperature (°C)
		Forward	Reverse	
SERPINE1	NM_001174114.2	TTCTGGTTGAAGGGCCAAGA	CATCTGCATCCTGAATTTCTCAA	60
PRKCA	XM_060395901.1	CCGACTTCATCTGGGGGTTT	CGTGTCGGGTCCTTTATCCG	60
PLG	XM_012183211.4	GTCCCGAGATGTACCACACC	ATATGGTGACGGCCACTGTC	60
NFKB1	XM_027970852.3	ACAGCAGATGGCCCATACCT	AGACGCCATCCTCACAATGTT	60
MTNR1A	NM_001009725.1	CGCAGGGAATGTGTTTGTGG	CTCAGGCTCCACCCATTGTT	60
LCN2	XM_042245651.2	CTGCAGTGCCTCCGGTC	AAGGGTCTCTGGGCTCTCTC	60
FGF7	NM_001009235.2	CCAGTTCCAGCAGGGAGATT	CATAAATGCGCAAGTCCAGC	60
ERBB2	XM_060394882.1	GATCCTCAAGGGAGGGGTCT	CCCAGCAGTTGGGAGCTTTA	60
CXCL8	NM_001009401.2	AGAGGTGTGCTTAGACCCCA	ACCCACACAGTACTCAAGGC	60
CXCL10	NM_001009191.1	GCACCTGCATCGAGATCAGTA	GCCTCTTTCTGTGTTCGAGGA	60
CDKN1A	NM_001161880.1	ACATTGACTATGTGCCAGGACC	AGGGGAAGGCGAGTTAGGTT	60
AREG	XM_042251881.2	CGCCATGACCTTCACAGCTA	TCTGATACCCTTCAGTTACGCT	60
β-actin	NM_001009784.4	CCCTGGAGAAGAGCTACGAG	GGTAGTTTCGTGAATGCCGC	60

**Table 2:** Effects of MT treatment on in vitro fertilization (IVF) efficiency of ovine oocytes collected via laparotomy.

Groups	Number of Inseminated Oocytes	Cleavage Rate (Mean ± SEM, %) ^a^	Total Blastocyst Rate (Mean ± SEM, %) ^b^
Control	400	250 (61.86 ± 4.23%) ^b^	77 (30.60 ± 3.67%) ^b^
20 mg MT	330	214 (64.92 ± 4.14%) ^b^	75 (35.97 ± 3.56%) ^b^
40 mg MT	578	461 (79.49 ± 4.96%) ^a^	223 (47.32 ± 2.80%) ^a^
80 mg MT	493	352 (72.31 ± 4.49%) ^b^	148 (40.30 ± 4.91%) ^b^

^a^ Cleavage rate = Number of cleaved oocytes/Total inseminated oocytes × 100%; ^b^ Total blastocyst rate = Total blastocyst count/Number of cleaved oocytes × 100%; Values labeled with different superscript lowercase letters (^a^, ^b^) within the same column indicate statistically significant differences (*p* < 0.05).

**Table 3:** Effects of MT treatment on morphological parameters of sheep ovaries.

Parameters	Control Group	40 mg MT Group	t-Value	*p*-Value
Length (mm)	16.92 ± 0.46	18.41 ± 0.48	2.232	0.035 *
Width (mm)	12.87 ± 0.49	14.01 ± 0.30	1.997	0.057
Thickness (mm)	9.31 ± 0.32	11.05 ± 0.35	3.699	0.001 **
Weight (g)	1.13 ± 0.08	1.49 ± 0.08	3.220	0.004 **
Volume (mm^3^)	1088.82 ± 81.88	1511.17 ± 99.06	3.286	0.003 **

Note: Data are mean ± SEM of bilateral ovarian means per ewe (n = 13 per group), compared by independent-samples *t*-tests. * *p* < 0.05, ** *p* < 0.01.

**Table 4:** Results of two-way repeated-measures ANOVA for serum reproductive hormones.

Hormone	Treatment (*p*-Value)	Time (*p*-Value)	Treatment × Time (*p*-Value)
MT	<0.0001 ****	<0.0001 ****	0.3687
E2	0.0020 **	0.2796	0.2185
IGF-1	0.0164 *	0.0029 **	0.0817
FSH	0.0567	0.2203	0.7534
LH	0.4178	0.7389	0.5808
P4	0.7193	<0.0001 ****	0.0037 **
GH	0.1204	0.0010 **	0.1434
PRL	0.6851	<0.0001 ****	0.0103 *
AMH	0.0795	0.2724	0.7778

Note: Data were analyzed by two-way repeated-measures ANOVA with treatment as a between-subject factor and time as a within-subject factor. Sphericity was assessed using Mauchly’s test, and the Greenhouse–Geisser correction was applied when sphericity was violated. *p*-values for treatment, time, and treatment × time interaction are shown. * *p* < 0.05, ** *p* < 0.01, **** *p* < 0.0001.

**Table 5:** Effects of melatonin (MT) treatment on embryo transfer outcomes in sheep.

Outcome	Control Group	40 mg MT Group	RR (95%CI)	Two-Sided Fisher’s Exact *p*-Value
Pregnancy rate	12/26 (46.2%)	15/27 (55.6%)	1.20 (0.70–2.06)	0.587
Lambing success among pregnant recipients	10/12 (83.3%)	14/15 (93.3%)	1.12 (0.84–1.49)	0.569

Note: Pregnancy rate was calculated as the number of pregnant recipients divided by the total number of ewes receiving embryo transfer. Lambing success was calculated as the proportion of pregnant recipients that subsequently delivered lambs. Relative risk (RR) was calculated using the control group as the reference category. Two-sided Fisher’s exact tests were used for between-group comparisons because of the limited sample size.

## Data Availability

The authors confirm that the original contributions presented in this study are reflected in this manuscript.

## Supplementary Materials

The following supporting information can be downloaded at: https://www.mdpi.com/article/10.3390/antiox15101346/s1. Supplementary Figure S1: Flow diagram showing animal allocation, analysis, and exclusion across experimental modules. Supplementary Table S1: Effect of melatonin on the number of various follicle types in ovine ovaries. Supplementary Table S2: Effects of melatonin on the expression of Ki-67, FSH-R, and other immunohistochemical indicators in ovarian tissues. Supplementary Figures S2–S5: Transcriptomic analysis results, including heatmap of DEGs, FPKM expression bar plot, and top 30 upregulated/downregulated GO enrichment maps (melatonin vs. control group). Supplementary Figures S6–S8: Donut charts showing the super-class, class, and sub-class distribution of differential metabolites (control vs. MT group).

## Abbreviations

The following abbreviations are used in this manuscript:

MT1	Melatonin Receptor 1
COCs	Cumulus-Oocyte Complexes
AOD	Average Optical Density
CIDR	Controlled Internal Drug Release
Ki-67	Marker of Proliferation Ki-67
FSH-R	Follicle-Stimulating Hormone Receptor
T-AOC	Total Antioxidant Capacity
MDA	Malondialdehyde
PRL	Prolactin
AANAT	Arylalkylamine N-Acetyltransferase
ASMT	Acetylserotonin O-Methyltransferase
PKA	Protein Kinase A
PCA	Principal Component Analysis
SIRT1	Sirtuin 1
DEG	Differentially Expressed Gene
TCA	Tricarboxylic Acid Cycle
HIF-1	Hypoxia-inducible Factor 1
AMH	Anti-Müllerian Hormone
HPO	Hypothalamic-pituitary-ovarian
Bcl-2	B-cell Lymphoma 2
GH	Growth Hormone
GSH	Glutathione
IGF-1	Insulin-like growth factor 1
