# Supplementary material for: Effects of Melatonin Dose Regimens on Follicular Development and Oocyte Developmental Competence in FSH-Superovulated Ewes: Redox and Molecular Responses
Source: Antioxidants (Basel). 2026 Oct 18;15(10):1346. doi: 10.3390/antiox15101346 (PMC13701921; doi:10.3390/antiox15101346)
Supplement: Supplementary file 1 [file antioxidants-15-01346-s001.zip › Supplementary File S1.pdf]

Supplementary Figures for Animal Allocation

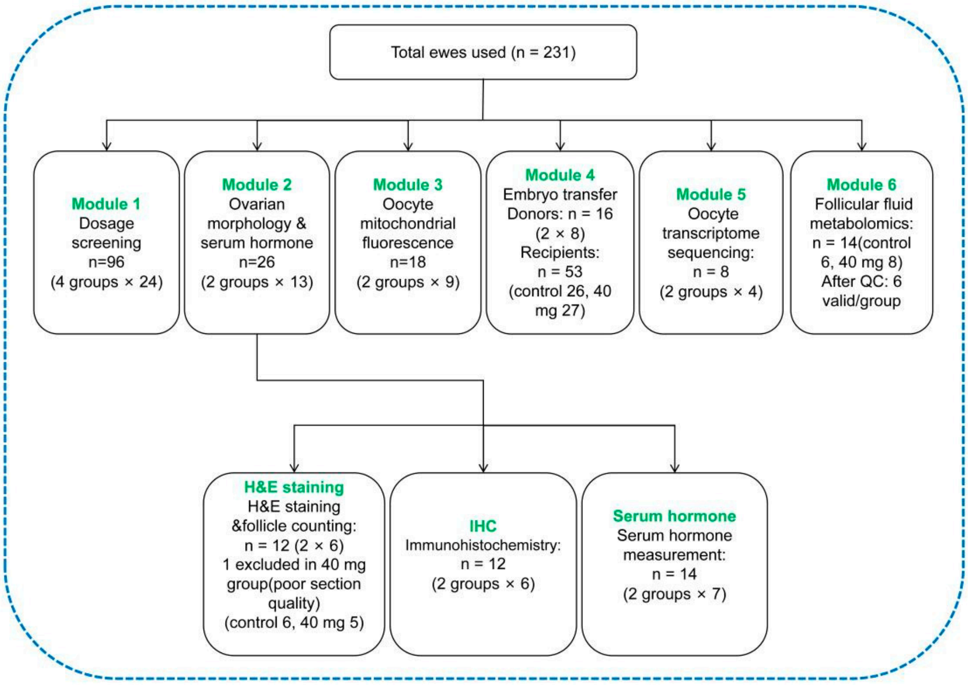

Figure S1. Flow diagram showing animal allocation, analysis, and exclusion across experimental modules.
