# Supplementary material for: Effects of Melatonin Dose Regimens on Follicular Development and Oocyte Developmental Competence in FSH-Superovulated Ewes: Redox and Molecular Responses
Source: Antioxidants (Basel). 2026 Oct 18;15(10):1346. doi: 10.3390/antiox15101346 (PMC13701921; doi:10.3390/antiox15101346)
Supplement: Supplementary file 1 [file antioxidants-15-01346-s001.zip › Supplementary File S2.pdf]

Supplementary Table S1. Effect of melatonin on the number of various follicle types in ovine ovaries

Follicle type	Control group (n = 6)	40 mg MT group (n = 5)	*p* value
Primordial follicle	68.25±17.98	92.40±13.63	0.2468
Primary follicle	6.58±2.06	15.57±2.78	0.0455*
Secondary follicle	3.00±2.03	2.67±0.71	0.3009
Atretic follicle	7.25±1.35	3.57±0.71	0.0447*
Total follicle	85.08±21.06	114.20±14.08	0.4286

Note: Data are presented as mean ± SEM. Control: n = 6 ewes; 40 mg MT: n = 5 ewes (one ewe in the 40 mg MT group was excluded due to poor section quality). Comparisons between groups were performed using Welch's t-tests for atretic follicles and Mann–Whitney U tests for primordial, primary, secondary, and total follicles, based on the normality of the data. *$p < 0.05$.
