# Supplementary material for: Effects of Melatonin Dose Regimens on Follicular Development and Oocyte Developmental Competence in FSH-Superovulated Ewes: Redox and Molecular Responses
Source: Antioxidants (Basel). 2026 Oct 18;15(10):1346. doi: 10.3390/antiox15101346 (PMC13701921; doi:10.3390/antiox15101346)
Supplement: Supplementary file 1 [file antioxidants-15-01346-s001.zip › Supplementary File S3.pdf]

Supplementary Table S2. Effects of melatonin on the expression of Ki-67, FSH-R and other immunohistochemical indicators in ovarian tissues

Protein	Detection Index	Control Group	40 mg MT Group	*p* value
Ki-67	Positive Cell Rate (%)	19.35±2.68	31.04±4.26	0.0470*
Ki-67	AOD	0.1169±0.0030	0.1215±0.0024	0.2597
FSH-R	AOD	0.1395±0.0035	0.1477±0.0043	0.0649
MT1	AOD	0.1316±0.0025	0.1658±0.0101	0.0188*
Bcl-2	AOD	0.1036±0.0036	0.1188±0.0075	0.1797
Bax	AOD	0.1032±0.0013	0.0894±0.0036	0.0260*
Bcl-2/Bax ratio	Ratio	1.007±0.0439	1.343±0.1104	0.0272*

Note: Data are mean ± SEM (n = 6 per group). Comparisons between groups were performed using Welch's t-tests for Ki-67 positive cell rate, Ki-67 AOD, MT1 AOD, and Bcl-2/Bax ratio, and using Mann–Whitney U tests for FSH-R AOD, Bcl-2 AOD, and Bax AOD, based on the normality of the data. AOD = average optical density. *$p < 0.05$.
