# Supplementary material for: Effects of Melatonin Dose Regimens on Follicular Development and Oocyte Developmental Competence in FSH-Superovulated Ewes: Redox and Molecular Responses
Source: Antioxidants (Basel). 2026 Oct 18;15(10):1346. doi: 10.3390/antiox15101346 (PMC13701921; doi:10.3390/antiox15101346)
Supplement: Supplementary file 1 [file antioxidants-15-01346-s001.zip › Supplementary File S4.pdf]

Supplementary Figures for Oocyte Transcriptomic Analysis

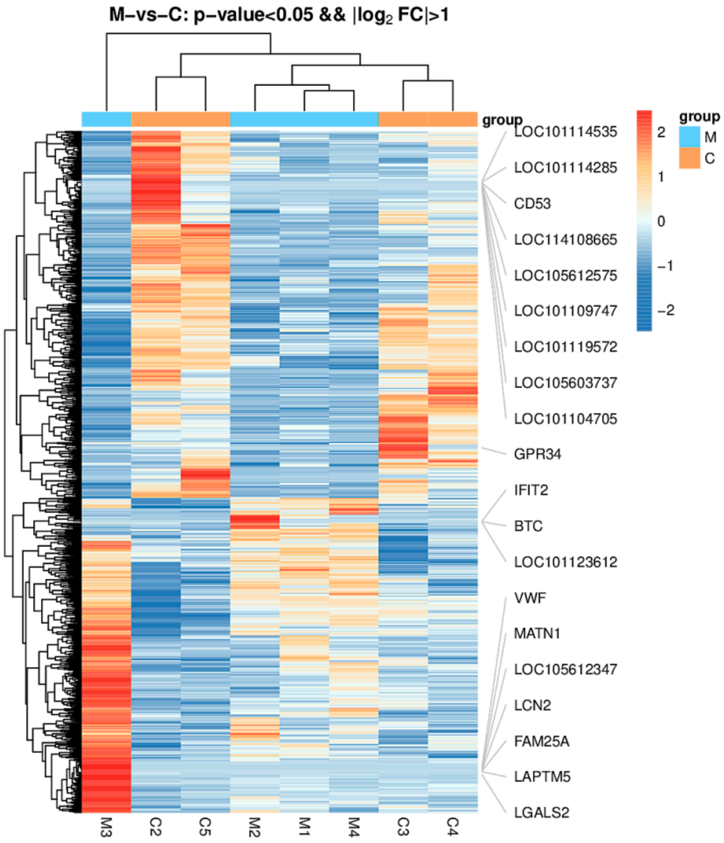

Figure S2. Heatmap clustering of differentially expressed genes (DEGs) in the melatonin group vs. the control group.

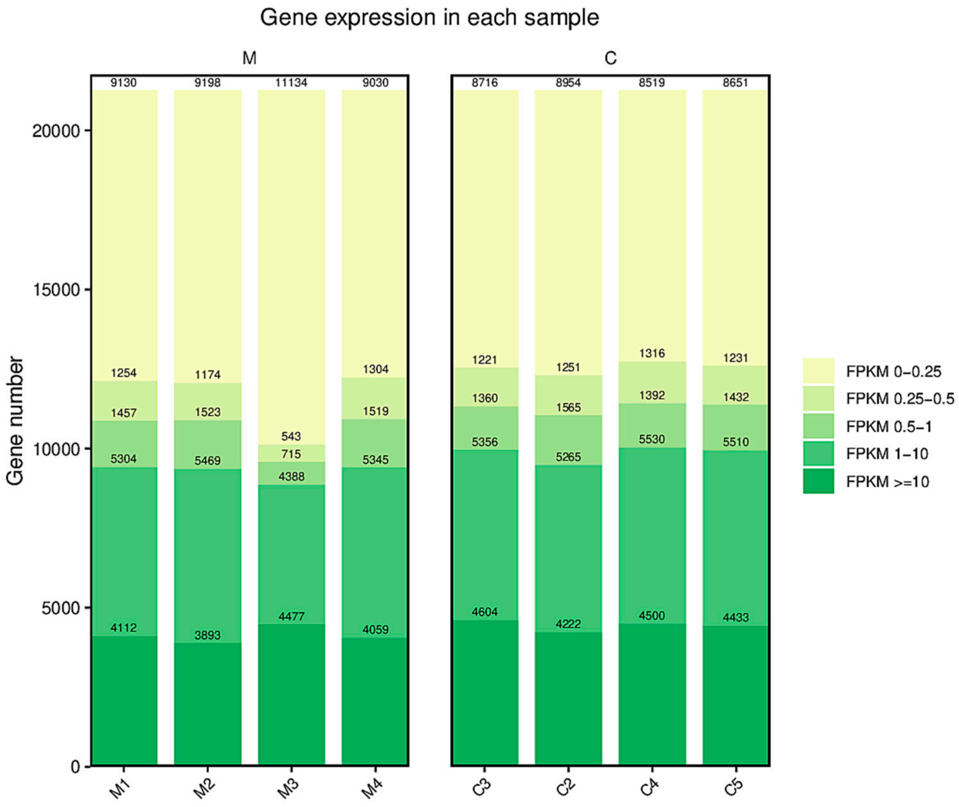

Figure S3. Bar plot of FPKM expression for individual samples from the melatonin and control groups.

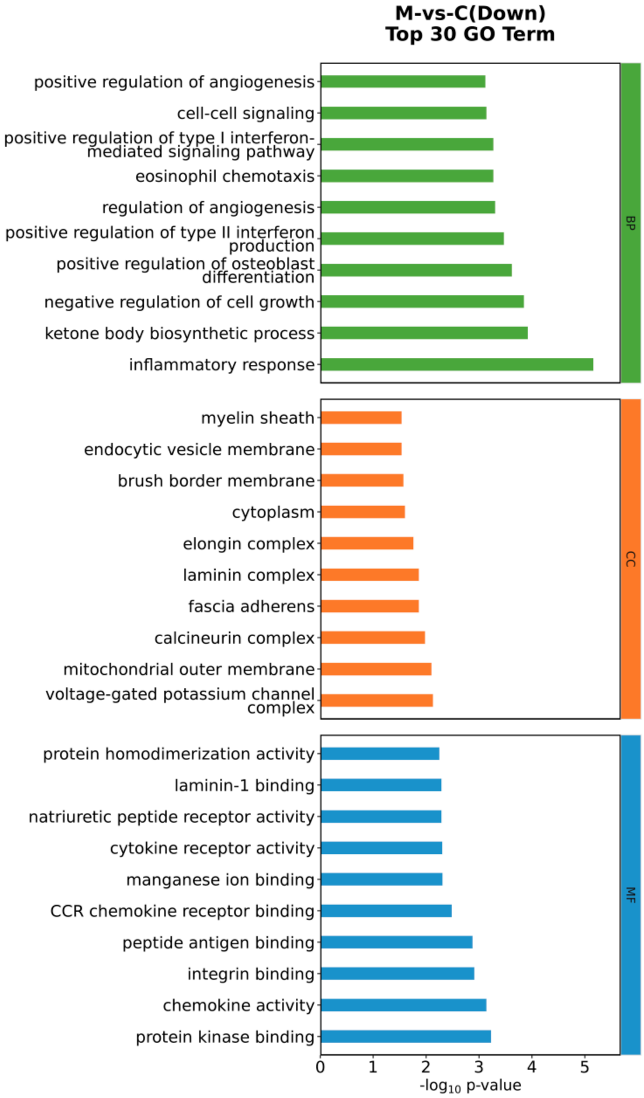

Figure S4. GO enrichment analysis of the top 30 downregulated genes.

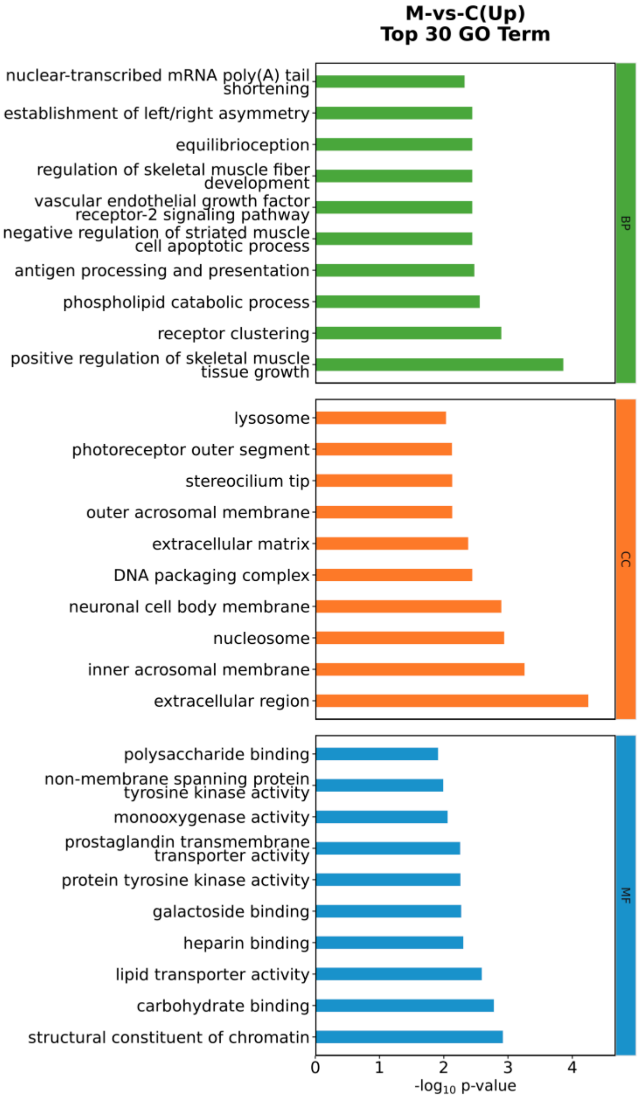

Figure S5. GO enrichment analysis of the top 30 upregulated genes.
