# Supplementary material for: Effects of Melatonin Dose Regimens on Follicular Development and Oocyte Developmental Competence in FSH-Superovulated Ewes: Redox and Molecular Responses
Source: Antioxidants (Basel). 2026 Oct 18;15(10):1346. doi: 10.3390/antiox15101346 (PMC13701921; doi:10.3390/antiox15101346)
Supplement: Supplementary file 1 [file antioxidants-15-01346-s001.zip › Supplementary File S5.pdf]

Supplementary Figures for Follicular Fluid Metabolomic Analysis

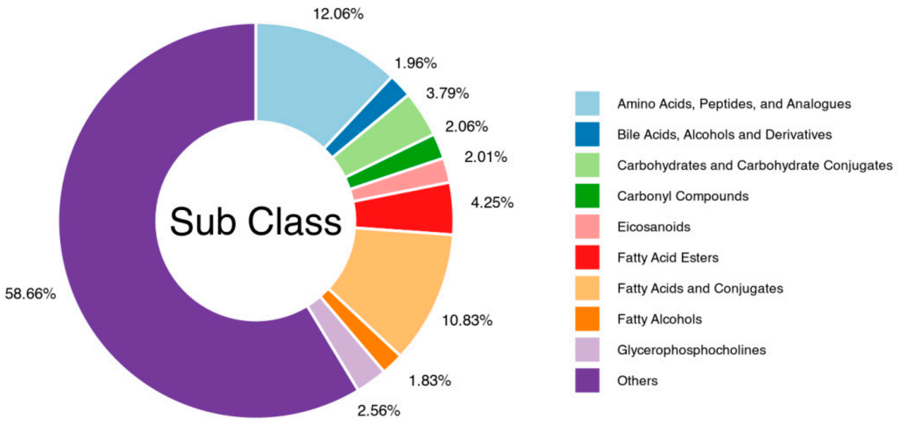

Figure S6. Donut chart showing the sub-class distribution of differential metabolites in the control group vs. the melatonin group.

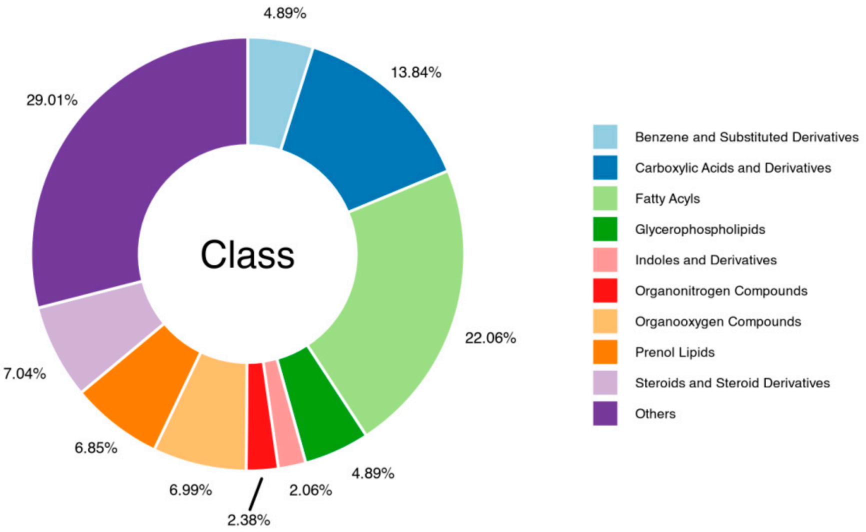

Figure S7. Donut chart showing the class distribution of differential metabolites in the control group vs. the melatonin group.

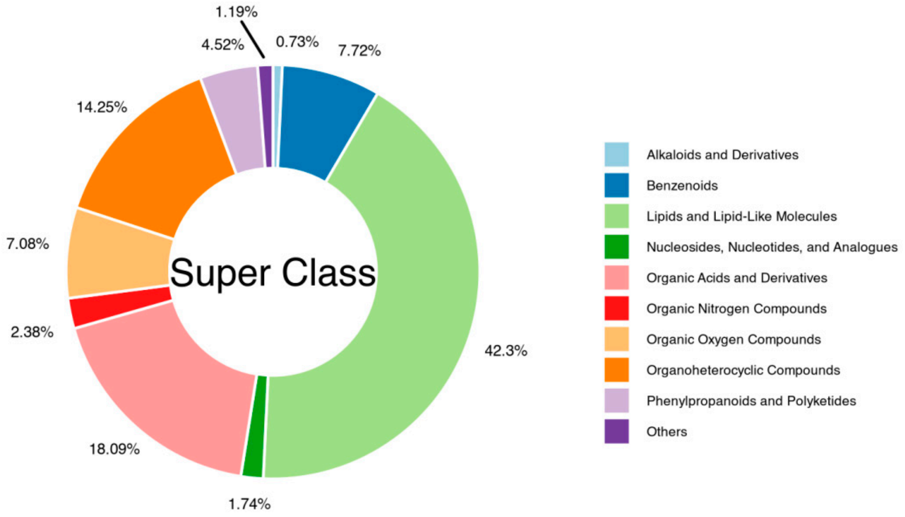

Figure S8. Donut chart showing the super-class distribution of differential metabolites in the control group vs. the melatonin group.
